# HISTONE DEACETYLASE19 Controls Ovule Number Determination and Transmitting Tract Differentiation

**DOI:** 10.1093/plphys/kiad629

**Published:** 2023-12-07

**Authors:** Silvia Manrique, Alex Cavalleri, Andrea Guazzotti, Gonzalo H Villarino, Sara Simonini, Aureliano Bombarely, Tetsuya Higashiyama, Ueli Grossniklaus, Chiara Mizzotti, Ana Marta Pereira, Silvia Coimbra, Subramanian Sankaranarayanan, Elisabetta Onelli, Simona Masiero, Robert G Franks, Lucia Colombo

**Affiliations:** Dipartimento di Bioscienze, Università degli Studi di Milano, Via Giovanni Celoria 26, Milan 20133, Italy; Dipartimento di Bioscienze, Università degli Studi di Milano, Via Giovanni Celoria 26, Milan 20133, Italy; Dipartimento di Bioscienze, Università degli Studi di Milano, Via Giovanni Celoria 26, Milan 20133, Italy; Department of Plant and Microbial Biology, North Carolina State University, Raleigh, NC 27606, USA; Department of Plant and Microbial Biology & Zurich-Basel Plant Science Center, University of Zurich, Zollikerstrasse 107, Zurich CH-8008, Switzerland; Dipartimento di Bioscienze, Università degli Studi di Milano, Via Giovanni Celoria 26, Milan 20133, Italy; Institute of Transformative Bio-Molecules (ITbM), Nagoya University, Furo-cho, Chikusa-ku, Nagoya, Aichi 464-8601, Japan; Department of Plant and Microbial Biology & Zurich-Basel Plant Science Center, University of Zurich, Zollikerstrasse 107, Zurich CH-8008, Switzerland; Dipartimento di Bioscienze, Università degli Studi di Milano, Via Giovanni Celoria 26, Milan 20133, Italy; Faculdade de Ciências da Universidade do Porto, Departamento de Biologia, Universidade do Porto, rua do Campo Alegre, Porto 4169-007, Portugal; LAQV Requimte, Sustainable Chemistry, Universidade do Porto, Porto 4169-007, Portugal; Faculdade de Ciências da Universidade do Porto, Departamento de Biologia, Universidade do Porto, rua do Campo Alegre, Porto 4169-007, Portugal; LAQV Requimte, Sustainable Chemistry, Universidade do Porto, Porto 4169-007, Portugal; Department of Biological Sciences and Engineering, Indian Institute of Technology Gandhinagar, Palaj, Gujarat 382355, India; Dipartimento di Bioscienze, Università degli Studi di Milano, Via Giovanni Celoria 26, Milan 20133, Italy; Dipartimento di Bioscienze, Università degli Studi di Milano, Via Giovanni Celoria 26, Milan 20133, Italy; Department of Plant and Microbial Biology, North Carolina State University, Raleigh, NC 27606, USA; Dipartimento di Bioscienze, Università degli Studi di Milano, Via Giovanni Celoria 26, Milan 20133, Italy

## Abstract

The gynoecium is critical for the reproduction of flowering plants as it contains the ovules and the tissues that foster pollen germination, growth, and guidance. These tissues, known as the reproductive tract (ReT), comprise the stigma, style, and transmitting tract (TT). The ReT and ovules originate from the carpel margin meristem (CMM) within the pistil. SHOOT MERISTEMLESS (STM) is a key transcription factor for meristem formation and maintenance. In all above-ground meristems, including the CMM, local *STM* downregulation is required for organ formation. However, how this downregulation is achieved in the CMM is unknown. Here, we have studied the role of HISTONE DEACETYLASE 19 (HDA19) in Arabidopsis (*Arabidopsis thaliana*) during ovule and ReT differentiation based on the observation that the *hda19-3* mutant displays a reduced ovule number and fails to differentiate the TT properly. Fluorescence-activated cell sorting coupled with RNA-sequencing revealed that in the CMM of *hda19-3* mutants, genes promoting organ development are downregulated while meristematic markers, including *STM*, are upregulated. HDA19 was essential to downregulate *STM* in the CMM, thereby allowing ovule formation and TT differentiation. *STM* is ectopically expressed in *hda19-3* at intermediate stages of pistil development, and its downregulation by RNA interference alleviated the *hda19*-3 phenotype. Chromatin immunoprecipitation assays indicated that *STM* is a direct target of HDA19 during pistil development and that the transcription factor SEEDSTICK is also required to regulate *STM* via histone acetylation. Thus, we identified factors required for the downregulation of *STM* in the CMM, which is necessary for organogenesis and tissue differentiation.

## Teaser

HISTONE DEACETYLASE19 regulates ovules and reproductive tract formation by repressing *SHOOT MERISTEMLESS* expression.

## Introduction

The number of seeds produced by a plant is important for the reproductive success of a species and, in the case of crops, for food security. Seed yield depends on several characteristics, like flower number, the number of ovules per pistil, as well as the capacity of the gynoecium to foster their fertilization, among others.

**Figure 1.**
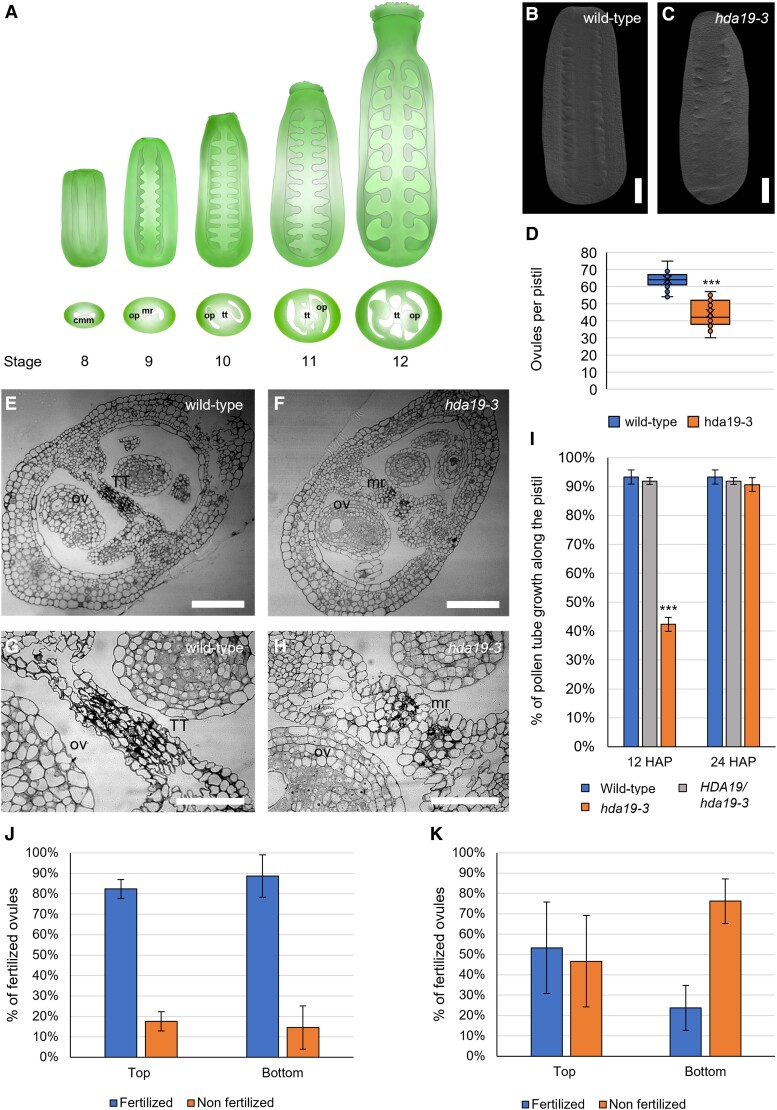
Ovule density and TT are abnormal in the *hda19-3* mutant. **A)** Scheme of the development of the ReT from the CMM from stages 8 to 12 of flower development according to Smyth et al. (1990). *Abbreviations:* CMM, carpel margin meristem; OP, ovule primordia; mr, medial ridge; TT, transmitting tract. **B, C)** Cleared pistils of the wild type **(B)** and *hda19-3***(C)** at stage 9, showing differences in ovule density. Images were digitally extracted for comparison. Scale bar = 50 *µ*m. **D)** Ovule number of wild-type (*n* = 70 pistils) and *hda19-3* (*n* = 85 pistils) plants. Center line = median; X = average; box limits = upper and lower quartiles; whiskers = 1.5× interquartile range; points = single measures and outliers. ***Student's *t*-test with *P*-value < 0.001. **E** to **H)** Transversal section of wild-type **(E, G)** and *hda19-3***(F**, **H)** pistils at stage 12. *hda19-3* shows unfused septum and defective TT due to the lack of degeneration of septum cells. Images **G** and **H** are close-ups of a portion of images **E** and **F**, respectively. *Abbreviations:* TT, transmitting tract; OV, ovule; mr, medial ridge. Scale bar = 50 *µ*m. **I)** Ratio of pollen tube length/pistil length at 12 and 24 HAP in wild-type (*n* = 8), heterozygous (*n* = 8), and homozygous (*n* = 12) *hda19-3* pistils, hand-pollinated with wild-type pollen. Bars represent the mean ± SE value. ***Student's *t*-test with *P*-value < 0.001, comparing wild type with *HDA19/hda19-3* or *hda19-3*. **J, K)** Seed set in the top half and bottom half of wild-type **(J)** and *hda19-3***(K)** siliques, hand-pollinated with wild-type pollen, showing that the bottom half of *hda19-3* siliques shows higher levels of unfertilized ovules than the top half. Bars represent the mean ± SE value of the percentage of unfertilized ovules in 10 wild-type and 10 *hda19-3* siliques.

The gynoecium or pistil contains the ovules and the tissues required for pollen germination, growth, and guidance. These tissues are known as the reproductive tract (ReT) and comprise the stigma, style, and transmitting tract (TT). Both structures emerge from the carpel margin meristem (CMM) (Reyes-Olalde et al. 2013; Chávez Montes et al. 2015), a determinate meristem that appears as a ridge in the medial part of the gynoecium at stage 7 of floral development (Fig. 1A; Smyth et al. 1990). As pistil development progresses, the CMM produces the ovules and the different structures composing the ReT (Fig. 1A). During stages 8 to 9, it produces the placenta and ovule primordia (OP), and the medial ridges fuse, forming the septum (Fig. 1A). In stages 10 to 12, all the organs complete their developmental program, and the gynoecium becomes competent for fertilization (Bowman et al. 1999; Roeder and Yanofsky 2006; Reyes-Olalde et al. 2013; Chávez Montes et al. 2015; Herrera-Ubaldo et al. 2019).

Ovule formation encompasses two steps: (1) boundary formation and primordium outgrowth and (2) identity determination. The first step is controlled by general modulators of lateral organ formation, such as *CUP-SHAPED COTYLEDON 1/2/3* (*CUC1/2/3*), *BLADE-ON-PETIOLE* (*BOP*), *PIN-FORMED 1* (*PIN1*), and *AINTEGUMENTA* (*ANT*) (Ishida et al. 2000; Mizukami and Fischer 2000; Vernoux et al. 2000; Norberg et al. 2005). Particularly, OP emerge from the placenta asynchronously. Indeed, ovules start to emerge from the placenta at stage 9a, while the final ovule number is reached only at stage 9c. This results in the presence of ovules of different sizes and developmental stages within the same pistil (Yu et al. 2020).

The second step is controlled by organ-specific genes. Ovule identity is redundantly determined by three MADS-domain transcription factors, SEEDSTICK (STK) and SHATTERPROOF1/2 (SHP1/2). In the respective triple mutant, ovules are replaced by carpelloid structures (Favaro et al. 2003; Pinyopich et al. 2003).

Regarding ReT tissues, the TT forms in the center of the septum from the top to the base of the pistil (Fig. 1A) (Crawford and Yanofsky 2008) and supports the growth of pollen tubes to reach the ovules. In Arabidopsis (*Arabidopsis thaliana*), pollen tubes grow through the intercellular spaces between TT cells, aided by the production of a growth-facilitating cell wall and the death of the TT cells. This process is developmentally regulated and starts before anthesis (Crawford and Yanofsky 2008; Pereira et al. 2021). The first stage of TT formation depends on transcription factors controlling the formation of the septum, such as ETTIN (ETT), SPATULA (SPT), SEUSS (SEU), ANT, and INDEHISCENT (IND) (Sessions and Zambryski 1995; Alvarez and Smyth 2002; Azhakanandam et al. 2008; Kay et al. 2013; Pereira et al. 2021). Later, genes like *NO TRANSMITTING TRACT* (*NTT*) and *HECATE1/2/3* (*HEC1/2/3*) control the maturation of TT cells (Gremski et al. 2007, Marsch-Martínez et al. 2014; Herrera-Ubaldo et al. 2019). Interestingly, STK and SHP1/2 are also required for TT formation in cooperation with NTT (Marsch-Martínez et al. 2014; Herrera-Ubaldo et al. 2019) and CESTA/HALF-FILLED (CES/HAF) (Di Marzo et al. 2020a, 2020b).

Genetic and transcriptomic analyses have revealed similarities between the CMM and undetermined meristems like the shoot apical meristem (SAM) (Wynn et al. 2011; Heidstra and Sabatini 2014; Gaillochet and Lohmann 2015; Villarino et al. 2016; Reyes-Olalde and de Folter 2019; Zúñiga-Mayo et al. 2019). However, how the activity of the CMM is controlled, leading to the consumption of the stem cells once organ formation is completed, is not known (Alvarez and Smyth 2002; Reyes-Olalde and de Folter 2019).

HISTONE DEACETYLASE 19 (HDA19) is a major regulator of the maintenance of the SAM (Long et al. 2006; Gorham et al. 2018; Chung et al. 2019), the flower meristem (Krogan et al. 2012; Bollier et al. 2018), and the root apical meristem (Pi et al. 2015). HDA19 is an epigenetic modifier that removes acetyl groups from histones, repressing the transcription of the associated genes (Kumar et al. 2021). HDA19 target specificity relies on the interaction with sequence-specific transcription factors and other cofactors (Kumar et al. 2021). For instance, in the inflorescence meristem (IM), HDA19 interacts with the auxin response factors ETT and AUXIN RESPONSE FACTOR 4 (ARF4) and the YABBY protein FILAMENTOUS FLOWERS (FIL) to downregulate the *KNOTTED-1-like* homeobox (*KNOX*) transcription factor gene *SHOOT MERISTEMLESS (STM)*, allowing flower primordia specification (Chung et al. 2019). Interestingly, it was previously suggested that STM's role is similar in the SAM and CMM (Scofield et al. 2007; Durbak and Tax 2011; Poulios and Vlachonasios 2018): *STM* has to be locally downregulated to allow the formation of organ primordia from the SAM (Long et al. 1996; Kanrar et al. 2006; Landrein et al. 2015). Likewise, in pistils, *STM* overexpression interferes with ovule identity determination and septum formation (Scofield et al. 2007).

Here, we show that mutations affecting *HDA19* lead to ovule number reduction and TT defects. We identified targets of HDA19 during ovule formation and TT differentiation through transcriptomic analysis of cells isolated by fluorescence-activated cell sorting (FACS), using a marker line active in TT, placenta, and ovules. We observed that *STM* was over- and ectopically expressed in TT and ovules in *hda19-3* compared to the wild type and that the *STM* locus showed more histone acetylation in *hda19-3* flowers. Our work also shows that the *stk* mutant has similar defects as *hda19-3* regarding ovule number and TT differentiation, and that STK binds the *STM* locus and regulates its acetylation state. Overall, our results indicate that the correct development of ovules and TT requires repression of *STM* by HDA19 and that STK is also necessary for histone acetylation-mediated downregulation of *STM* in the CMM.

## Results

### Development of the ReT requires HDA19

Several histone deacetylases (HDAs) are important for plant development (Kumar et al. 2021). HDA6, HDA9, and HDA19 are partially redundant for reproductive traits like flowering time (Kang et al. 2015; Ning et al. 2019), but HDA19 mutants are the only ones that show a reduced seed set (Kang et al. 2015; Ning et al. 2019). Although this phenotype was previously reported (Zhou et al. 2013; Kuhn et al. 2020), its molecular basis has not been investigated. Here, we focus on the role of HDA19 during ovule number determination and TT differentiation as prefertilization characters contributing to the final seed number.

For that, we analyzed ovule number in wild type and *hda19-3* flowers. We observed that, although *hda19-3* pistils are similar in size (Fig. 1, B and C), they contain significantly fewer ovules (51 ± 3 ovules) than the wild type (67 ± 4 ovules) (Fig. 1D), indicating a lower ovule density. Next, we analyzed pistil radial organization, observing that, in *hda19-3* flowers, the medial ridges remain unfused at stage 12 (Fig. 1, E to H). Also, the degeneration of the subepidermal cells required for TT maturation is drastically reduced (Fig. 1, F and H) in comparison to the wild type (Fig. 1, E and G). These defects impair pollen tube growth inside the pistil (Fig. 1I; Supplemental Fig. S1) and reduce seed set predominantly in the bottom half of the silique (Fig. 1, J and K). In hand-pollination experiments using wild-type pollen, the percentage of unfertilized ovules in wild-type siliques at 16 h after pollination (HAP) was low and similar in the top and bottom halves of the siliques (Fig. 1J). In *hda19-3* flowers, fertility is overall lower compared to the wild type (Fig. 1, J and K), as noted before (Zhou et al. 2023). However, the percentage of unfertilized ovules in the bottom half of the siliques was notably higher with respect to the top half (Fig. 1K). Altogether, we conclude that HDA19 is required for proper OP specification and TT development.

### Transcriptomic analysis of *hda19-3* sorted cells supports the role of HDA19 in ReT development and ovule number determination

*HDA19* is highly expressed in most tissues of the inflorescence (Zhou et al. 2005). As we wanted to focus on its role during ovule number determination and TT differentiation, we designed a strategy for the isolation of these specific cell types for transcriptomic analysis.

We crossed *hda19-3* with a line containing the genomic region containing *STK* fused to the gene encoding the Green Fluorescent Protein (pSTK::STK-GFP, Mizzotti et al. 2014), driving the expression of STK-GFP in the placenta, ovules, and TT from stage 8 to 12 (Mizzotti et al. 2014; Herrera-Ubaldo et al. 2019). After verifying that the spatial expression pattern of STK-GFP was equivalent in both genetic backgrounds (Supplemental Fig. S2), we collected whole inflorescences, except open flowers, directly in a solution (Villarino et al. 2016) to produce protoplast. GFP-positive protoplasts were then collected by FACS, and RNA was extracted to identify differentially expressed genes by RNA-seq.

Enrichment of *STK-GFP* in sorted protoplasts was validated by RT-qPCR (Supplemental Fig. S3) before library preparation. Three and four samples of RNA from GFP-positive cells (80 to 100,000 cells/replicate) from wild-type and *hda19-3* plants, respectively, were used for library preparation and RNA Illumina sequencing (HiSeq2500).

**Figure 2.**
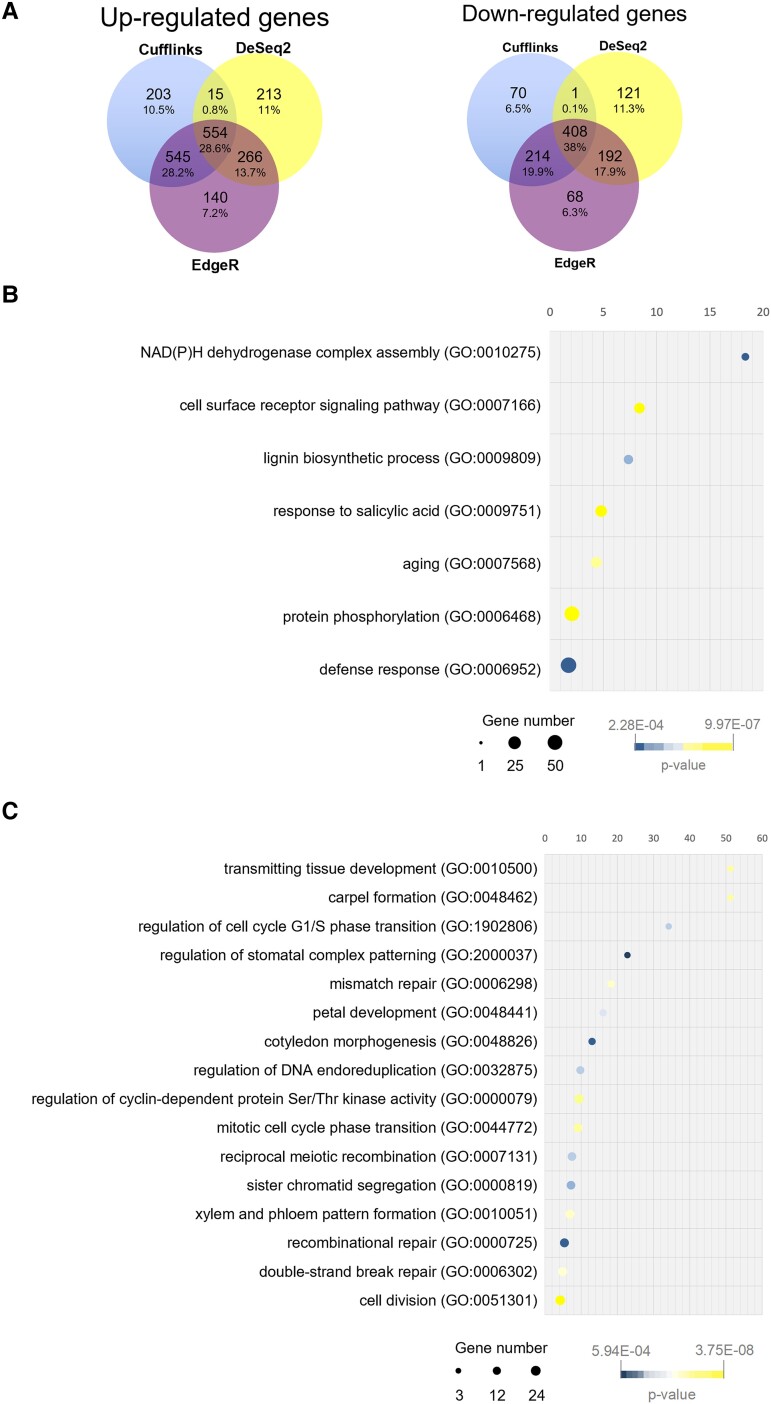
Reproductive tract transcriptomic comparison of wild-type and *hda19-3* flowers. **A)** Venn diagrams showing the overlap of up- and downregulated genes (FDR < 0.001 and logFC > |1.5|) detected by Cufflinks, EdgeR, and DeSeq2. **B)** Enrichment analysis of “Biological Process” GO terms associated with upregulated genes (logFC > |1.5|) detected by all three programs. **C)** Enrichment analysis of “Biological Process” GO terms associated with downregulated genes (logFC > |1.5|) detected by all three programs (fold enrichment > 4). Graphs show overrepresented child-most (tip) GO terms. Parent GO terms (root terms) have not been plotted for simplicity. Enrichment analysis was performed using Panther (Mi et al. 2018, 2019) with the default parameters set by the program.

Since HDA19 is an epigenetic regulator, we used a stringent approach to classify differentially expressed genes (DEGs). We analyzed the RNA-seq data using Cufflinks (Trapnell et al. 2012), DeSeq2 (Love et al. 2014), and EdgeR (Robinson et al. 2009) (Supplemental Data Set 1 to S3). Then, we compared the DEGs and selected only those common in all three analyses (Supplemental Data Set 4). This approach identified 554 up- and 408 downregulated genes (Fig. 2A).

To survey the roles of DEGs, we performed a gene ontology (GO) enrichment analysis of “Biological Process’ terms associated with the up- and downregulated genes, respectively (Fig. 2, B and C; Supplemental Data Set 5 and 6), using Panther (Mi et al. 2018, 2019). Among the upregulated genes, we found the enriched GO terms “response to salicylic acid”, “defense response”, and “aging”, which reflect previously described roles of had19 (Zhou et al. 2005; Kim et al. 2008; Tanaka et al. 2008; Zhu et al. 2010; Choi et al. 2012) (Fig. 2B; Supplemental Data Set 5). Likewise, “NAD(P)H dehydrogenase complex assembly” relates to the role ofHDA19 in regulating the light response (Benhamed et al. 2006; Guo et al. 2008; Jan et al. 2011).

Among the downregulated genes, the most enriched GO terms (Fig. 2C) were “transmitting tissue development” and “carpel formation”, with a fold enrichment of 51.28 (Fig. 2C; Supplemental Data Set 6).

Overall, the enrichment analysis of GO terms showed that genes associated with known roles of HDA19 were present among the DEGs. Moreover, genes necessary for the normal progression of carpel and TT development were downregulated in *hda19-3* sorted cells, supporting the validity of our dataset and sampling strategy.

### Meristematic markers and genes involved in ovule and TT development are deregulated in the *had19-3* mutant

**Figure 3.**
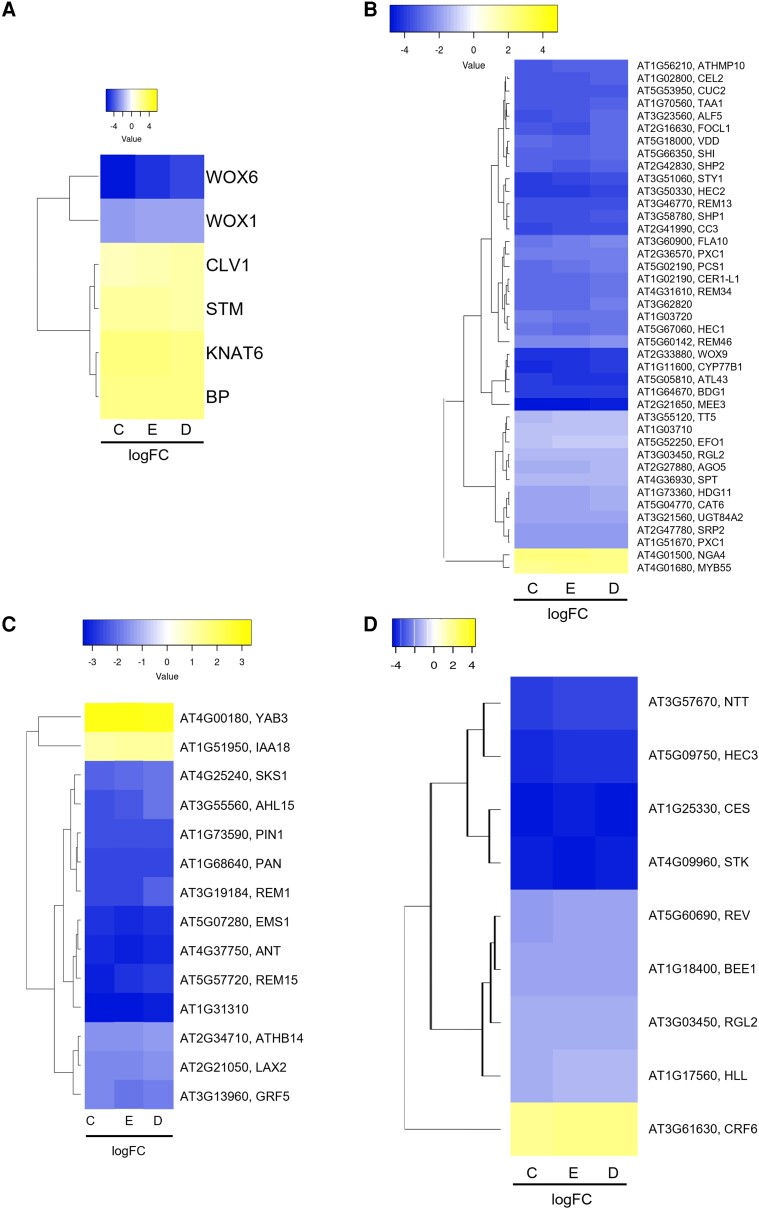
CMM-specific genes are deregulated in the *hda19-3* mutant. **A)** Meristem marker genes deregulated in the *hda19-3* mutant. **B)** CMM-specific genes from stages 6 to 8 of pistil development according to Villarino et al. (2016) that are deregulated in the *hda19-3* mutant. **C)** CMM-specific genes from stages 8 to 10 of pistil development according to Wynn et al. (2011) that are deregulated in *hda19-3.***D)** Notable ovule number and TT regulators are deregulated in *hda19-3*. Genes plotted correspond to genes with an FDR < 0.001 and logFC > |1.2| in the RNA-Seq data according to Cufflinks (C), EdgeR (E), and DeSeq2 (D). Genes were hierarchically clustered by average clustering using Euclidean distances. Heatmaps were obtained using Heatmapper (http://heatmapper.ca/). The scales are relative to the maximum and minimum values of each table and represents the logFC (wild-type vs *hda19-3*) as detected by each program.

HDA19 regulates the maintenance of stem cell populations in several meristems (Long et al. 2006; Krogan et al. 2012; Pi et al. 2015; Bollier et al. 2018). Therefore, we checked for deregulated meristematic markers in our dataset, and we found that *CLAVATA1* (*CLV1*), as well as the KNOX genes *STM*, *KNAT1/BREVIPEDICELLUS* (*BP*) and *KNAT6*, showed higher expression in *hda19-3* compared to wild-type sorted cells (Fig. 3A).

*STM*, *BP*, and *KNAT6* are partially redundant for SAM maintenance (Byrne et al. 2002), and their local downregulation at the boundaries of the meristem is necessary for organ initiation (Belles-Boix et al. 2006; Ragni et al. 2008; Zhao et al. 2015). Therefore, their upregulation in *hda19-3* plants could hinder the formation of CMM-derived structures.

Furthermore, to identify CMM-specific genes associated with ReT differentiation and ovule initiation, we cross-referenced our dataset with two pre-existing sets of CMM-specific genes (Wynn et al. 2011; Villarino et al. 2016). We found that several genes involved in TT and style development like *HEC1*, *HEC2*, *SPT*, and *STYLISH* (*STY1*) (Sohlberg et al. 2006; Gremski et al. 2007; Nahar et al. 2012; Kuhn et al. 2020) were downregulated in *hda19-3* plants (Fig. 3B), possibly explaining the defects in the TT (Fig. 1, E to H) and style (Kuhn et al. 2020). Additionally, several well-known players of ovule initiation, such as *PHABULOSA* (*PHB*), *ANT*, *PERIANTHIA* (*PAN*) and *PIN1*, were downregulated in the *hda19-3* mutant (Fig. 3C), consistent with their reduced number of ovules (Fig. 1D).

Only four CMM-specific genes were upregulated in *hda19-3*: *NGATHA4* (*NGA4*) and *MYB55* (Villarino et al. 2016) (Fig. 3B); and *YABBY3* (*YAB3*) and *INDOLE-3-ACETIC ACID INDUCIBLE 18 (IAA18)* (Wynn et al. 2011) (Fig. 3C). *NGA4* and *YAB3* are negative regulators of lateral organ formation (Alvarez et al. 2009; González-Reig et al. 2012; Lee et al. 2015), consistent with the ReT phenotype of *hda19-3* plants. The role of *IAA18* and *MYB55* in the pistil is not known, but *iaa18* mutants have reduced fertility (Uehara et al. 2008), and *MYB55* is a direct target of BRASSINAZOLE-RESISTANT 1 (BZR1) (He 2005), participating in organ boundary establishment in the SAM (Bell et al. 2012; Gendron et al. 2012). Therefore, the upregulation of these genes might also interfere with ReT development.

Finally, we checked the expression of some additional regulators of TT and ovule number determination that were not included in these two datasets (Wynn et al. 2011; Villarino et al. 2016) due to their experimental conditions. For example, *HEC3* and *STK* itself were downregulated (Fig. 3D). It is worth noting that, although *STK* shows a log fold-change (logFC) close to −4 in *hda19-3* mutants, its spatial expression pattern was unchanged with respect to the wild type (Supplemental Fig. S2).

Overall, analysis of CMM-specific genes revealed that several positive regulators of TT differentiation and ovule initiation were downregulated (*PIN 1, ANT*), while *KNOX* genes involved in meristem maintenance (*STM*, *BP*, *KNAT6*) and negative regulators of lateral organ development (*NGA4, YAB3*) were upregulated in *hda19-3* sorted cells. As HDA19 is a transcriptional repressor, their upregulation suggests that HDA19 might directly regulate meristematic genes and/or genes involved in inhibiting the formation and differentiation of CMM-derived structures.

### *STM* is ectopically expressed and over-acetylated in the ReT of the *hda19-3* mutant

Among the possible candidates contributing to *hda19-3*'s defects in ovule formation and TT differentiation (Fig. 1), we focused our attention on *STM*, which was upregulated in *hda19-3* mutants compared to the wild type (Fig. 3A). HDA19 downregulates *STM* in the IM to allow organ development from this meristem (Chung et al. 2019). Therefore, we hypothesized that a similar situation could occur in the CMM: as *STM* is required for pistil development (Scofield et al. 2007), HDA19 may also regulate it there.

**Figure 4.**
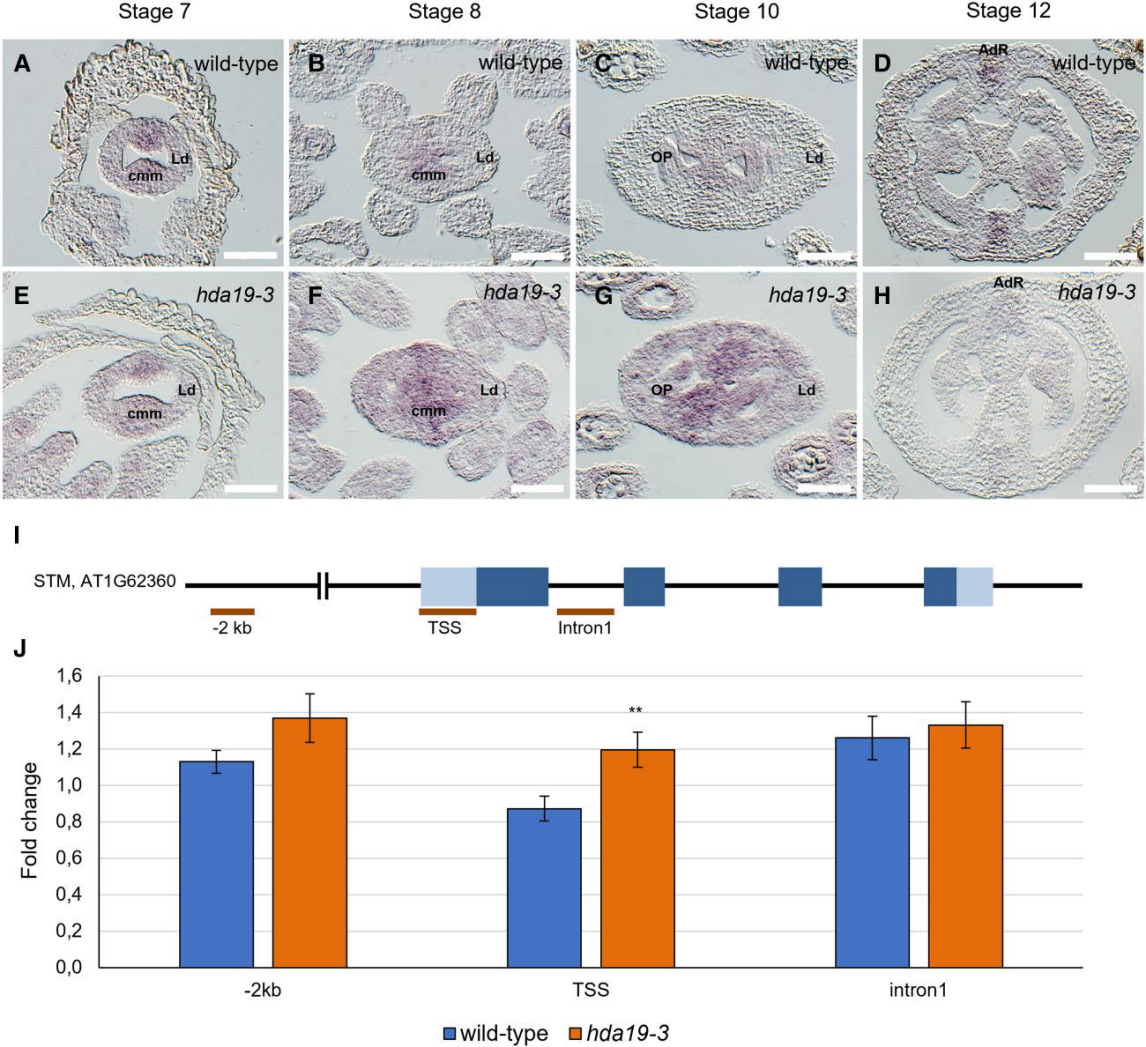
*STM* is ectopically expressed and highly acetylated in *hda19-3* pistils. **A** to **H)** Detection of *STM* mRNA by ISH in wild-type **(A** to **D)** and *hda19-3***(E** to **H)** pistils. At stage 6, *STM* mRNA is specifically expressed in the CMM of wild-type pistils **(A, B)**. Then expression decreases during the following stages until it is restricted to the adaxial replum zone **(C, D)**. Instead, in the *hda19-3* mutant during stages 7 to 10 when ovule primordia arise, the expression of *STM* increases and expands outside the CMM **(F, G)**, to finally become restricted to the replum later on **(H)**. *Abbreviations:* CMM, carpel margin meristem; LD, lateral domain; OP, ovule primordia; AdR, adaxial replum. Scale bar = 50 *µ*m. **I)** Regions of the *STM* locus tested for changes in H3K9/14ac are indicated by bars. **J)** H3K9/14ac ChIP-qPCR of *STM* locus in wild-type and *hda19-3* flowers. Graph shows the mean ± SD result of *STM* regions enrichment in the *hda19-3* background with respect to the wild type, among three independent biological replicates of the H3K9/K14ac ChIP-qPCR experiment performed on wild-type, *hda19-3, stk*, and *stkshp1shp2* flowers. **Student's *t*-test with *P*-value < 0.01.

We investigated the spatial expression pattern of *STM* in wild-type and *hda19-3* pistils by in situ hybridization (ISH) using an *STM*-specific antisense probe (Fig. 4, A to H). At stage 7, the expression pattern of *STM* was similar in both genotypes (Fig. 4, A and E). However, from stages 8 to 10, the expression of *STM* in the medial ridge increased in *hda19-3* and expanded to the placenta, OP, and even the lateral domain (Fig. 4, B and C, F and G). After stage 10, *STM* expression became restricted to the adaxial replum zone in both genotypes (Fig. 4, D and H). *STM* promotes meristematic fate; therefore, ectopic expression in the placenta, OP, and TT from stages 8 to 10 might interfere with ovule initiation and TT development, causing the phenotypes observed in the *hda19-3* mutant.

To analyze whether the changes in *STM*'s expression pattern could be caused by variations in histone acetylation levels resulting from the absence of HDA19, we collected the inflorescence apex (including flowers up to stage 12 but excluding the IM) from wild-type and *hda19-3* plants and performed ChIP-qPCR. ChIP-qPCR is a widely used technique to reveal differences in the levels of specific histone modifications at a specific locus (Saleh et al. 2008; Petrella et al. 2020). We used an antibody against HISTONE 3 (H3) acetylated at lysine 9 and 14 (K9/14) (H3K9/14ac) (Fig. 4, I and J) to capture DNA fragments associated with acetylated histone H3. Since no other histone de/acetylases are substantially deregulated in *hda19-3* sorted cells (Supplemental Fig. S4), the changes in *STM* histone acetylation observed with this antibody should be caused mostly by the direct action of HDA19.

We tested the acetylation levels in several regions of the *STM* locus: (1) the distal promoter (−2 kb) (Fig. 4I) because it was reported that HDA19 can deacetylate these regions of the *STM* locus (Chung et al. 2019); (2) the transcription start-site (TSS), as its acetylation level generally is associated with active gene expression (Wang et al. 2017; Kim et al. 2021; Kumar et al. 2021); and (3) the first intron. We observed a significant increase in acetylation at the *STM* TSS in *hda19-3* and a moderate increase at the promoter region, while no notable increment in acetylation was found in the first intron (Fig. 4J). In conclusion, our results suggest that, at the stages tested, HDA19 is required for the deacetylation of the *STM* locus. Moreover, this acetylation pattern differs from that of Chung et al. (2019), suggesting that HDA19 acts on different regions in each meristem.

### Downregulation of *STM* alleviates ReT defects of the *hda19-3* mutant in an expression level-dependent manner

To verify if the ectopic expression of *STM* was involved in the reduction of ovule number and TT defects in the *hda19-3* mutant, we generated an RNA interference (*RNAi*) construct to reduce *STM* levels in the *hda19-3* background. We used the pFRH vector as, in our experience, it causes a moderate downregulation of the target gene (Fornara et al. 2004) that is compatible with the objective of our experiment.

**Figure 5.**
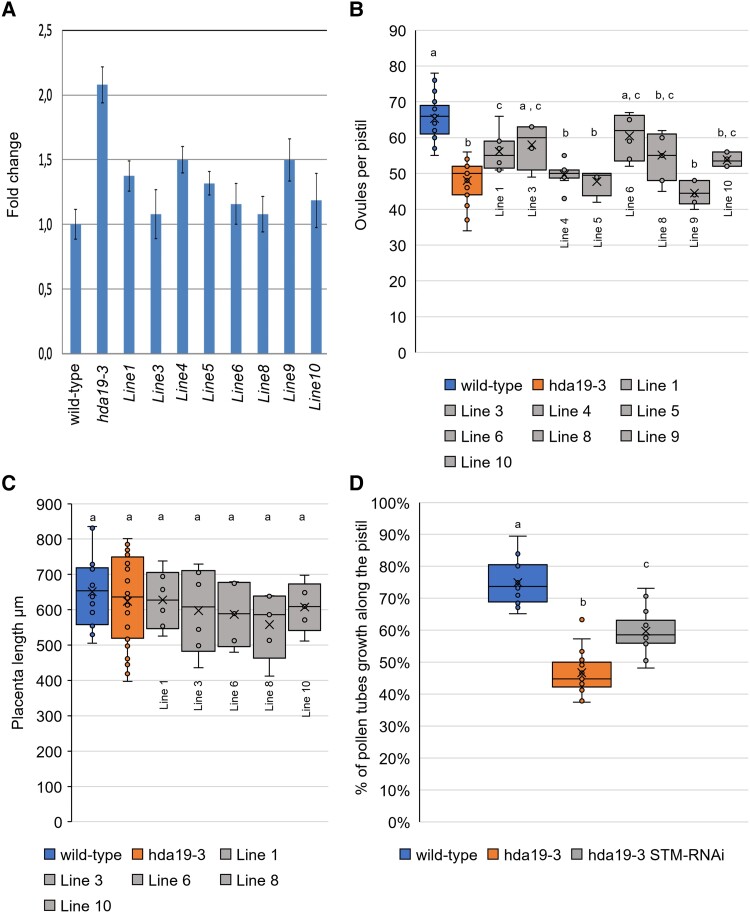
Downregulation of *STM* alleviates *hda19-3* ReT phenotypes. **A)** Level of *STM* mRNA in inflorescences of wild-type, *hda19-3*, and eight independent *hda19-3 STMRNAi* lines. Error bars represent the mean ± SE among three replicates. **B)** Ovule number per pistil of wild-type (*n* = 25), *hda19-3* (*n* = 27) and *hda19-3 STMRNAi* lines (in order from Line 1 to Line 10: *n* = 8, 4, 10, 4, 6, 7, 6, 6). Center line = median; X = average; box limits = upper and lower quartiles; whiskers = 1.5× interquartile range; points = single measures and outliers. The statistical significance of differences in ovule number was determined by a one-way ANOVA and a Bonferroni post hoc test for multiple comparisons. Letters over boxes denote significantly different averages (*P* < 0.05), comparing wild type, *hda19-3* and *hda19-3 STMRNAi* lines. **C)** Placenta length of wild-type, *hda19-3,* and *hda19-3 STMRNAi* lines that showed significant changes in ovule number (Lines 1, 3, 6, 8, 10). Center line = median; X = average; box limits = upper and lower quartiles; whiskers = 1.5× interquartile range; points = single measures and outliers. The statistical significance of differences in placenta length (*P* < 0.05) was determined by a one-way ANOVA with a Bonferroni post hoc test for multiple comparisons among wild-type, *hda19-3* and *hda19-3 STMRNAi* lines. **D)** Pollen tube growth measured as pollen tube length/total length of the pistil. Pollen tubes were allowed to grow for 14 HAP of emasculated flowers. Growth was visualized by aniline-blue staining and the lengths of pollen tubes and pistils were measured using ImageJ. Wild-type (*n* = 17 pistils); *hda19-3* (*n* = 17 pistils); *hda19-3 STMRNAi* (pool of Lines 1 and 10) (*n* = 18 pistils). Center line = median; X = average; box limits = upper and lower quartiles; whiskers = 1.5× interquartile range; points = single measures and outliers Letters over boxes indicate statistical differences as determined by a one-way ANOVA (*P* < 0.05) and a Bonferroni post hoc test for multiple comparisons.

We analyzed eight independent *hda19-3* lines transformed with *p35S::STM-RNAi::pFRH* (Fig. 5; Supplemental Fig. S5). We characterized the T1 generation to avoid random silencing of the construct in subsequent generations. We measured *STM* transcript levels in wild-type, *hda19-3*, and *hda19-3 p35S::STM-RNAi::pFRH* (*hda19-3 STMRNAi* hereafter) inflorescences by RT-qPCR. The levels of *STM* transcript in the *hda19-3* mutant were twice those in the wild type (Fig. 5A), and all the *hda19-3 STMRNAi* transformants showed *STM* mRNA levels in between those of *hda19-3* and the wild type (Fig. 5A).

Ovule number and density increased in the *hda19-3 STMRNAi* plants according to the level of *STM* downregulation (Fig. 5B), suggesting that the *STM* expression level in the gynoecium is related to ovule number determination. Lines 4 and 9 showed the mildest reduction of *STM* levels (ca. 75% of *hda19-3* levels) and, in accordance, did not show significant changes in ovule number (Fig. 5B). Line 5 was an exception, as it showed a reduction in *STM* levels similar to line 1 (ca. 35% reduction) (Fig. 5A), but while Line 1 showed an increase in ovule number, Line 5 did not (Fig. 5B). As expected, placenta length remained unchanged in all inspected lines (Fig. 5C). Therefore, the increase in ovule number represents an increase in ovule density (Fig. 5, B and C).

We selected Lines 1 and 10 for the inspection of TT phenotypes as they had comparable *STM* expression levels and ovule density, and flowers were still available after the experiments shown in Fig. 5, A and B.

Although the septum of Lines 1 and 10 was still partially unfused (Supplemental Fig. S5, A to C), the ridges were contacting more closely (Supplemental Fig. S5, B and C), and the subepidermal cells exhibited a wild-type-like phenotype (Supplemental Fig. S5, A to C). In addition, hand-pollination experiments revealed an improved pollen tube growth rate in the transgenic lines compared to the *hda19-3* mutant (Fig. 5D; Supplemental Fig. S5, D to F). We hand-pollinated wild-type, *hda19-3,* and *hda19-3 STMRNAi* pistils (Lines 1 and 10) with wild-type pollen and stained them with aniline blue 14 HAP (Fig. 5D; Supplemental Fig. S5, D to F). In a preliminary experiment, we determined that pollen tubes reach the bottom of wild-type pistils 16 HAP. Therefore, at 14 HAP, we would be able to precisely quantify the differences between each genotype. At 14 HAP, pollen tubes grew through 70% of wild-type pistils and 60% of *hda19-3 STMRNAi* pistils, a significant increase with respect to *hda19-3*, where pollen tubes barely grew 40% of the pistil length (Fig. 5D; Supplemental Fig. S5, D to F). Overall, even if the anatomical changes in the TT were mild (Supplemental Fig. S5, A to C), they improved pollen tube growth in comparison to the *hda19-3* mutant.

In conclusion, our results strongly suggest that ectopic expression of *STM* (Fig. 4, A to H) contributes to the ReT phenotypes present in the *hda19*-3 mutant (Fig. 1) because reducing *STM* expression in *hda19-3* to wild-type levels led to a recovery of ovule number and density (Fig. 5, B and C) and of TT function (Fig. 5D; Supplemental Fig. S5, D to F).

### *Stk* mutants show defects in the ReT and increased histone acetylation at the *STM* locus

HDA19 does not directly bind DNA; therefore, it has to be recruited to DNA by DNA-binding proteins to regulate its targets. In the IM, HDA19 interacts with ETT, ARF4, and FIL (Chung et al. 2019) to downregulate *STM*. However, in the pistil, ETT, ARF4, and FIL are mainly expressed in the lateral domains at stages 8 and 9 (Sessions et al. 1997; Pekker et al. 2005; González-Reig et al. 2012). Consequently, the action of HDA19 in the CMM is probably mediated by a different complex.

Among the transcription factors that are co-expressed with *HDA19* in the CMM, we highlight STK as a possible candidate for several reasons. STK controls several aspects of ovule development (in redundancy with SHP1/2) (Favaro et al. 2003; Pinyopich et al. 2003) and is required for septum and TT development (Colombo et al. 2010; Herrera-Ubaldo et al. 2019; Di Marzo et al. 2020a, 2020b) Moreover, STK shows repressor activity associated with histone deacetylation of its direct targets (Mizzotti et al 2014), suggesting it might act in conjunction with HDAs.

**Figure 6.**
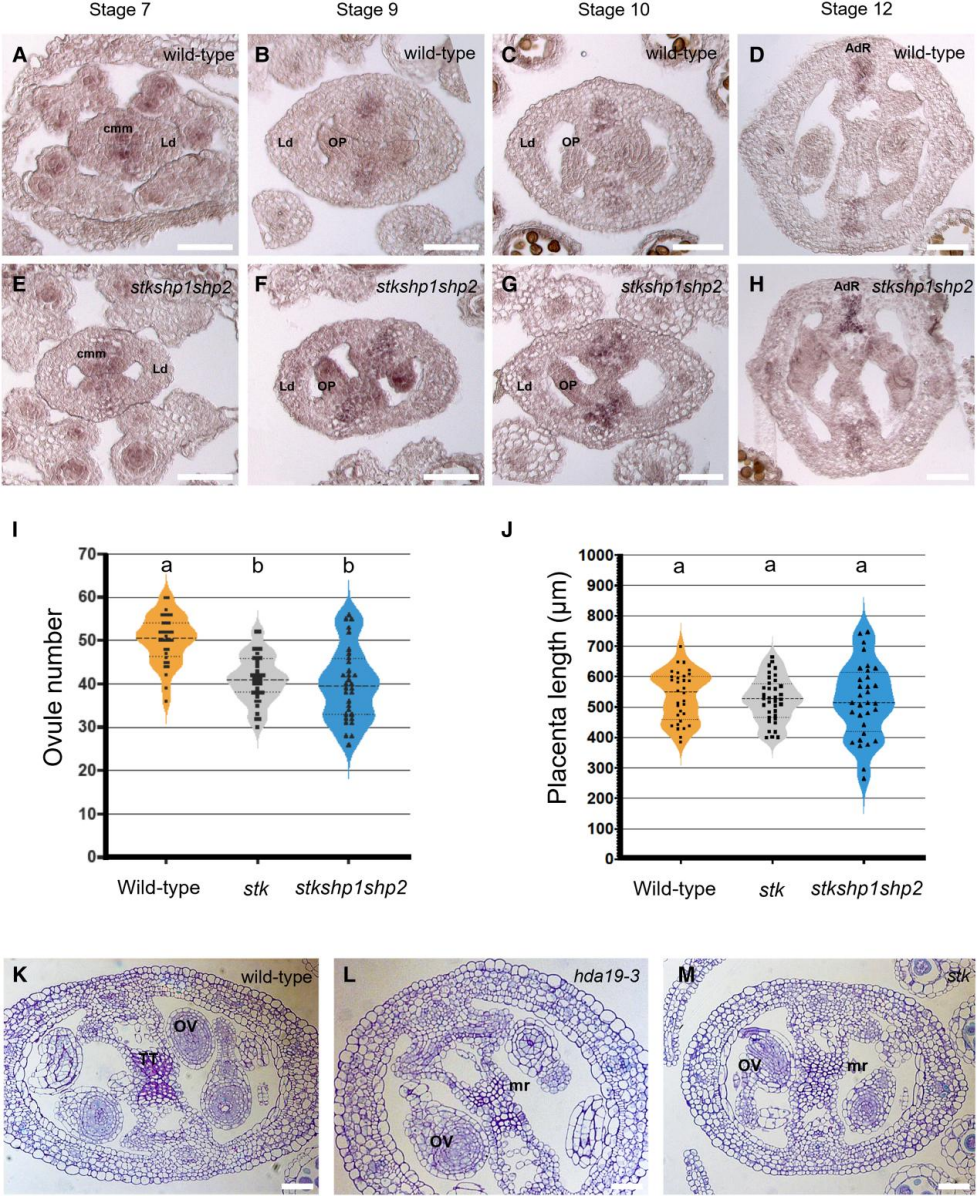
STK regulates *STM* expression in the CMM. **A** to **H)** Detection of *STM* mRNA in the wild type **(A** to **D)** and the *stk shp1 shp2* triple mutant **(E** to **H)** by ISH. *STM* is over- and ectopically expressed in the *stk shp1 shp2* triple mutant at stages 7 to 10 of pistil development **(F, G)** as compared to the wild type **(B, C)**. *Abbreviations:* CMM, carpel margin meristem; LD, lateral domain; OP, ovule primordia; AdR, adaxial replum. Scale bar, 50 *µ*m. **I, J**) Ovule number **(I)** and placenta length **(J)** in wild type (*n* = 31)*, stk (n = 35),* and *stk shp1 shp2 (n = 31)*. Center dashed line = median; Upper and lower dotted lines = upper and lower quartiles; Violin ends = 1.5× interquartile range; Squares or triangles within the violins = single measures and outliers Letters over violin-plots indicate statistical differences as determined by a one-way ANOVA (*P* < 0.05) and a Bonferroni post hoc test. **K–M)** Transversal sections of stages 10 to 11 wild-type **(K)**, *hda19-3***(L)**, and *stk* pistils **(M)**. *hda19-3* and *stk* pistils show defective subepidermal cells in the TT. *Abbreviations:* TT, transmitting tract; OV, ovule; mr, medial ridge. Scale bar, 50 *µ*m.

To investigate if *STK* has a role in the regulation of *STM* in the CMM, we performed ISH using an *STM*-specific antisense probe (Fig. 6, A to H). To account for the possible redundancy with SHP1/2, we probed *stk shp1 shp2* triple mutant pistils. *STM* was ectopically and overexpressed in *stk shp1 shp2* (Fig. 6, E to H), in a similar fashion to what we had previously observed in the *hda19-3* mutant (Fig. 4, A to H). This observation suggests that *STK* or *SHP1/2* could be involved in *STM* regulation during ReT development.

Then, we measured ovule number and placenta length: the *stk* mutant showed a reduced ovule number (Fig. 6I) without changes in placenta length (Fig. 6J), similar to *hda19-3* mutants (Fig. 1). The *stk shp1 shp2* triple mutant did not show statistically significant additive effects (Fig. 6, I and J), suggesting that SHP1 and SHP2 are not involved in ovule number reduction.

Finally, we evaluated the TT morphology in stk pistils. While ridges were fused as previously described (Di Marzo et al. 2020a, 2020b), we found that the subepidermal cells looked similar to those in *hda19-3* mutants (Fig. 6, K to M).

### STK directly binds the *STM* locus and is involved in its deacetylation

**Figure 7.**
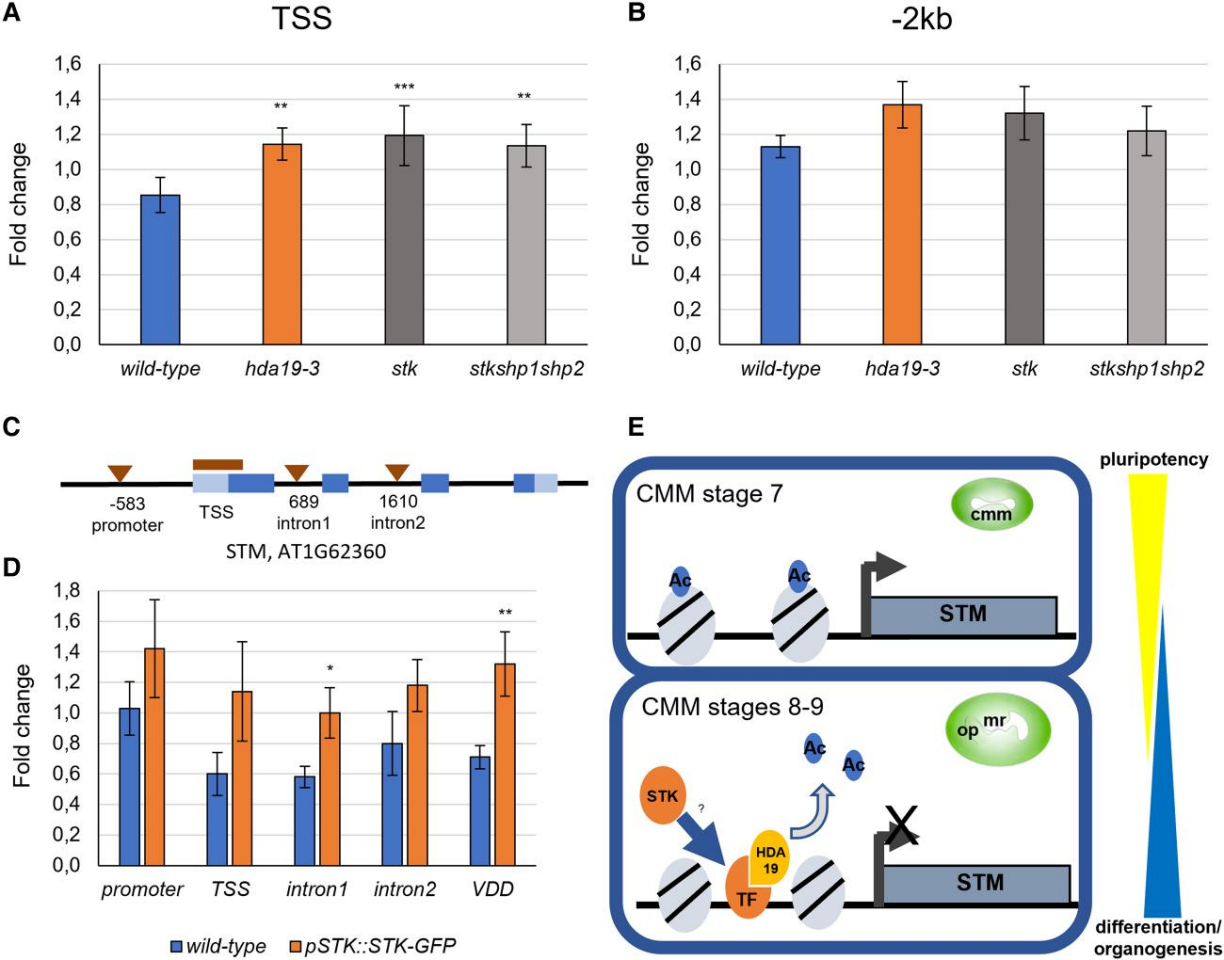
STK binds to the *STM* locus. **A, B)** H3K9/14ac ChIP-qPCR of *STM* TSS **(A)** and of a distal promoter region (−2 kb) **(B)** in the wild type, *hda19-3*, *stk*, and *stk shp1 shp2* mutants. The pattern of acetylation at the *STM* locus is similar among the different mutant genotypes. Graphs show the mean ± SD value of enrichment among three independent biological replicates of the H3K9/14ac ChIP-qPCR experiment performed on wild-type, *hda19-3, stk*, and *stk shp1 shp2* flowers. Student's *t*-test, **P-value < 0.01. ***P-value < 0.001. **C)** Regions of the *STM* locus tested by anti-GFP ChIP-qPCR in wild-type and *pSTK::STK-GFP* flowers. Triangles mark CaRG boxes detected by AthaMap (Steffens et al. 2004) while the bar marks the TSS region of *STM*. **D)** ChIP-qPCR to test binding of STK (*pSTK::STK-GFP*) to the promoter, TSS, first intron, and second intron of *STM*. *VDD* is a known direct target of STK, so it was used as positive control. Graph shows the mean ± SD enrichment value among four independent biological replicates of the anti-GFP ChIP-qPCR experiment. Student's *t*-test, *P-value < 0.05. **P-value < 0.01. **E)** Model of the regulation of *STM* expression in the CMM involving HDA19 and STK. Until stage 7, the expression of *STM* in the CMM is high due to the presence of acetylated histones in association with the *STM* locus. At stages 8 to 9, when the CMM starts producing the ReT organs, HDA19 deacetylates the histones associated with the *STM* locus, leading to its downregulation and thereby allowing the formation of OP and TT. Direct binding of STK to the *STM* locus facilitates the action of HDA19, potentially by generating multimeric complexes involved in HDA19 binding and stabilization to the *STM* locus. *Abbreviations and symbols:* CMM, carpel margin meristem; mr, medial ridge; OP, Ovule primordia; TF, transcriptional factor; Ac, acetyl group.

To explore if STK was required to regulate the acetylation state of the *STM* locus in the CMM, we performed ChIP-qPCR using an anti H3K9/14ac antibody on inflorescences (including flowers up to stage 12 but excluding the IM) of wild-type, *hda19-3, stk,* and *stk shp1 shp2* plants (Fig. 7, A and B), analyzing the same regions tested in *hda19-3* mutants (Fig. 4I). Similar to what we had observed in *hda19-3, stk* and *stk shp1 shp2* mutants showed an increase in H3K9/14ac at the level of the TSS, and a moderate increase at the promoter (Fig. 7, A and B). This observation supports the hypothesis that HDA19 and STK might cooperatively regulate the acetylation state of these regions.

To provide further evidence, we verified whether STK could directly bind to the *STM* genomic region by performing ChIP-qPCR on inflorescences (including flowers up to stage 12 but excluding the IM) of wild-type and *pSTK::STK-GFP* plants using an anti-GFP antibody. We found one MADS-domain DNA-binding motif (a CaRG box) in the proximal promoter area and two in the first and second introns of the *STM* locus (Fig. 7C). In addition to those, we also tested the region around the TSS of *STM* that was enriched in H3K9/14ac (Fig. 7A). As a positive control, we used *VERDANDI* (*VDD*), a direct target of STK (Matias-Hernandez et al. 2010) (Fig. 7D). The first *STM* intron showed the strongest enrichment, followed by the TSS. In contrast, the second intron and the promoter showed no enrichment (Fig. 7D). This result indicates that STK can directly bind the *STM* locus and that its action is required to regulate its expression level (Fig. 6, A to H).

Taken together, our phenotypic analysis, expression studies, and ChIP-qPCR experiments show that *HDA19* and *STK* activity have similar effects on the *STM* expression domain and transcript level. Their activity is required to regulate CMM functionality and to allow ReT development and ovule number determination, both essential traits for plant reproductive fitness (Fig. 7E).

## Discussion

Here, we show that *HDA19* is necessary for ovule initiation and TT differentiation (Fig. 1; Supplemental Fig. S1). To pinpoint the role of *HDA19* in these tissues, we performed an RNA-seq of *pSTK::STK-GFP*-expressing cells isolated by FACS from wild-type and *hda19-3* backgrounds. We found that several genes involved in meristem maintenance were upregulated in *hda19-3* mutant ReTs (Fig. 3). We focused on studying *STM* because of its well-known role in meristematic activity (Barton and Poethig 1993; Clark et al. 1997) and the recent report that HDA19 is necessary to downregulate *STM* in the IM (Chung et al. 2019). Other upregulated *KNOX* genes like *BP* (Byrne et al. 2002) were less attractive as, in the pistil, BP is associated with the differentiation of the replum (Alonso-Cantabrana et al. 2007; González-Reig et al. 2012), rather than the ovules and TT. *CLV1* was another potential target of HDA19, but the simultaneous upregulation of *CLV1* and *STM* suggested that the upregulation of *CLV1* could be a consequence of the spatial and temporal expansion of the meristematic activity of *hda19-3* CMMs (Fig. 3), rather than its cause, as *STM* is downregulated in *clv1* mutants (Durbak and Tax 2011). Nevertheless, it is still possible that these genes play a role together with *STM* in mediating the CMM phenotypes of the *hda19-3* mutant, and it will be interesting to study their contribution in the future. Instead, genes involved in organogenesis like *SPT*, *CUC1/2*, *ANT*, *PAN*, *PIN1, HEC1* or *NTT* were downregulated in sorted cells of *hda19-3* plants (Fig. 3), suggesting that they are not directly regulated by HDA19.

Loss-of-function *stm* mutants lack a functional SAM and, consequently, fail to produce flowers. Therefore, to assess whether *STM* deregulation was involved in the ReT phenotypes of *hda19-3*, we reduced *STM* levels in *hda19-3* plants by RNAi using a weak constitutive promoter. The downregulation of *STM* in the *hda19-3* background partially restored ovule number and density and pollen tube growth in the TT (Fig. 5; Supplemental Fig. S5). None of the transgenic lines displayed a complete rescue of the *hda19-3* phenotype. However, this is not surprising given that HDA19 regulates many genes, so it is unlikely that ectopic expression of *STM* is the only cause of all of *hda19-3*'s ReT defects. So, after all, downregulation of *KNAT6* and *BP*, partially redundant with *STM* in the SAM (Byrne et al. 2002), might be necessary for a complete rescue.

Previously, Scofield and collaborators observed that overexpressing *STM* through an inducible system led to the transformation of ovules into carpelloid structures (Scofield et al. 2007). This aligns with our observations but represents a more extreme phenotype, probably because the expression levels of *STM* achieved by their strategy were much higher than those observed in *hda19-3*, leading to a strong enough downregulation of *STK* and *SHP1/2* to cause the conversion of ovules into carpelloid structures (Scofield et al. 2007).

Our results support the idea that the CMM functions in a similar way as its indeterminate counterparts like the SAM or IM (Long et al. 2006; Chung et al. 2019) and provide insights into how its activity is terminated. However, this aspect requires further research, as ISH showed that, from stage 10, *STM* expression in the *hda19-3* mutant is similar to that in the wild type (Fig. 4, A to H). This finding suggests that, in addition to HDA19, other factors contribute to *STM* silencing in the CMM since lack of HDA19 delays *STM* silencing but does not prevent it completely.

Then, as HDA19 is unable to bind DNA on its own, we searched for partners that might mediate its interaction with DNA. We turned our attention to STK, based on its expression in the TT, placenta, and OP (Supplemental Fig. S2), its known roles in ovule and TT development (Pinyopich et al. 2003, Herrera-Ubaldo et al. 2019, Di Marzo et al. 2020a, 2020b), and that fact that the repression of some of its targets is associated with changes in histone acetylation (Mizzotti et al. 2014). The existence of similar defects in CMM-derived organs (Figs. 1 and 6, I to M), and a similar histone acetylation pattern at the *STM* locus in *hda19-3* and *stk* mutants (Figs. 4, I, J and 7, A, B), supports STK as a player required for *STM* regulation by HDA19 in the CMM. Nevertheless, it should be noted that, while the *stk* mutant shows a reduction of ovule number and density similar to *hda19-3*, the TT defects are milder in *stk* than in *hda19-3* plants. The role of *STK* in the development of the TT was already described (Marsch-Martínez et al. 2014; Herrera-Ubaldo et al. 2019, Di Marzo et al. 2020a, 2020b), but single *stk* mutants showed very mild defects that became more severe when *stk* was combined with the *ntt* and *ces* mutants. Overall, this suggests that, in the TT, HDA19 might act in cooperation with additional proteins to regulate *STM*.

Mutant analysis indicated that *SHP1/2* are not involved in this process. Although *STK* was first described as a member of the ovule identity complex redundant with *SHP1/2* (Favaro et al. 2003; Pinyopich et al. 2003), they also have nonredundant functions during reproductive development (Liljegren et al. 2000; Pinyopich et al. 2003; Mizzotti et al. 2012, 2014; Ezquer et al. 2016; Di Marzo et al. 2020a, 2020b). Our results suggest that the function in the CMM is another instance of the specific roles of the three paralogs.

Finally, we found that STK binds the *STM* locus at the level of the TSS and the first intron (Fig. 7, C and D). MADS-domain proteins form tetramers that are able to bind two DNA sequences simultaneously, creating DNA loops (Smaczniak et al. 2012). Based on this and the fact that the TSS shows a higher acetylation level in both mutants, a plausible model is that a MADS-domain complex containing STK and HDA19 binds to the *STM* locus, forming a loop between the TSS and the first intron. This would facilitate HDA19's deacetylation of the TSS region of *STM,* thereby reducing its expression and allowing organ initiation from the CMM (Fig. 7E).

It would have been interesting to study the mode of interaction between STK and HDA19. However, the fact that only repressed direct targets of STK are regulated through histone deacetylation (Mizzotti et al. 2014) suggests that STK must be able to form different multimeric complexes comprising different proteins. In other words, not every complex containing STK contains HDA19. This suggests that the mechanism of interaction between STK and HDA19 is either indirect (e.g. through other MADS-domain proteins) or regulated by post-translational modifications or alternative splicing.

SUMOylation is an interesting candidate modification to mediate the interaction of HDA19 and STK. HDA19 and other proteins of its core complex, like TOPLESS, are SUMOylated (Miller et al. 2010, 2013), while STK has predicted SUMO-interacting motifs (Zhao et al. 2014). When a protein is SUMOylated, usually, only a small fraction of the existing protein (5% to 10%) is modified (Xiao et al. 2015). Therefore, at a given time, only the HDA19-SUMOylated fraction would interact with STK, explaining the simultaneous action of STK as an activator (non-HDA19 interacting) and as a repressor (interacting with SUMOylated HDA19). Another possibility is alternative splicing. STK has six different splicing forms that differ in the presence/absence of several N-terminal exons. Therefore, it is possible that not all proteins derived from alternative splicing are able to interact with HDA19. Overall, this prompted us to discard the idea of testing the interaction between STK and HDA19 in the current work, as it would represent a project on its own.

An interesting hypothesis derived from our work is that *STK* expression at stage 8 (Pinyopich et al. 2003), before OP start to form, might be necessary to reduce *STM* levels (Fig. 7C) to allow OP initiation and septum fusion. This is supported by the observation that *STM* levels are normal in *hda19-3* mutants before stage 8 (Fig. 4, A to H), suggesting that HDA19 regulates *STM* during stages 8 to 10 when *STK* starts to accumulate.

Finally, we performed FACS-coupled transcriptomics of gynoecium cells without inducible systems, avoiding variability and artifacts reported previously (Villarino et al. 2016). In addition, our dataset containing TT, placenta, and sporophytic ovule cells will be highly useful for future studies of pistil and ovule development. Indeed, we already uncovered possible roles of genes like *YAB3* or *MYB55*. For example, *YAB3,* which represses medial factors (González-Reig et al. 2012) and mutations thereof affect CMM development, was proposed to act noncell autonomously as ISH did not reveal expression in the CMM (Wynn et al. 2011). As *pSTK::STK-GFP* is not expressed in the lateral domain, our data indicates that, in the wild-type, *YAB3* is indeed expressed in the middle domain at low levels that might be undetectable by ISH.

In conclusion, our results show that the tight control of *STM* levels achieved through the action of HDA19 and STK is essential to maximize the reproductive fitness of the plant, as even moderate changes in its expression lead to defects in the ReT that reduce seed set.

## Materials and methods

### Plant materials and growth conditions

Plants for phenotypic analyses were grown in a greenhouse under long-day conditions (16 h light, 8 h dark) at 22 to 24 °C. For cell sorting experiments, plants were grown in a walk-in growth chamber under continuous light at 22 °C.

Arabidopsis (*A. thaliana)* wild-type Col-0 seeds were available in the laboratory. *hda19-3* (SALK_139445) was provided by the Nottingham Arabidopsis Stock Center (NASC). The *pSTK::STK-GFP* line was available in the laboratory (Mizzotti et al. 2014).

*stk* and *STK/stk shp1 shp2* seeds (used to obtain the *stk shp1 shp2* triple mutant by segregation) were previously described and available in the laboratory (Favaro et al. 2003; Pinyopich et al. 2003).

### Ovule number and placenta length measurement

Inflorescences were fixed overnight in ethanol:acetic acid 9:1 and rehydrated in an ethanol series. Pistils (stages 7 to 10) were dissected and mounted in chloral hydrate:glycerol:water (8:1:3, w/v/v) and immediately observed under a Zeiss Axiophot D1 microscope (Carl Zeiss MicroImaging) equipped with DIC optics and an Axiocam MRc5 camera (Zeiss) with Axiovision software (version 4.1). Ovules were counted manually. Placenta length was measured using the Axiovision software. For statistical analysis in Fig. 1 wild type and *hda19-3* were confronted by Student's *t*-test (two-tailed distribution, homoscedastic). For experiments in Figs. 5 and 6 the statistical significance of differences in ovule number and placenta length was determined by a one-way ANOVA and a Bonferroni post hoc test for multiple comparisons (*P* < 0.05).

### Pistil histology and imaging

Individual wild type, *hda19-3, p35SRNAi:STM*, and *stk* flowers were fixed in 2% (w/v) paraformaldehyde and 2.5% (w/v) glutaraldehyde in PIPES buffer [0.025 M, pH 7, 0.001% (v/v) Tween-80], placed under vacuum for 1 h, and then stored at 4 °C overnight. The material was dehydrated in an ethanol series and embedded in LR White resin. Thick sections (500 *µ*m) were obtained with a Leica EM UC7 Ultramicrotome, stained with 1% (w/v) toluidine blue (Sigma-Aldrich, St Louis, MO, USA), and mounted with DPX (Sigma). Slides were observed under a Zeiss AxioImager AZ microscope equipped with a Zeiss Axiocam MRc3 camera using Zen Imaging software (Zen 2011 SP1).

### Aniline blue staining for pollen tube growth analysis

Flowers were emasculated and pollinated with wild-type pollen. Pollen tubes were allowed to grow for different times depending on the experiment (12, 14, 16 or 24 HAP). Then, pistils were fixed overnight in 9:1 ethanol:acetic acid, washed three times with water, incubated in 1 M NaOH overnight, washed three times in water, and incubated in 0.1% aniline blue (w/v). Pistils were imaged using a Zeiss AxioPhot D1 microscope equipped with fluorescence filters. Images were recorded with an Axiocam MRc5 camera (Zeiss) with Axiovision software (version 4.1). Pollen tube length was measured using ImageJ (Schneider et al. 2012). For experiments reported in Fig. 1 and Supplemental Fig. S1, statistical significance was determined by Student's *t*-test (two-tailed distribution, homoscedastic), comparing wild type with HDA19/*hda19-3* or *hda19-3*. For the experiment in Fig. 5 and Supplemental Fig. S5, statistical significance was determined by a one-way ANOVA and a Bonferroni post hoc test for multiple comparisons (*P* < 0.05).

### Protoplast recovery and fluorescence activated cell sorting

The *pSTK::STK-GFP* construct was introgressed into the *hda19-3* background and then used for cell sorting, along with the parental *pSTK::STK-GFP* line. *pSTK::STK-GFP* is expressed specifically in the TT, placenta, and ovules during stages 8 to 12 (Mizzotti et al. 2014). To be able to isolate sufficient numbers of cells expressing STK-GFP in a short time, we collected whole inflorescences, except for open flowers, directly in the solution used for protoplasting (Villarino et al. 2016).

Protoplasting and sorting were performed as described (Villarino et al. 2016), with some modifications. Namely, cells were sorted into RLT buffer from the RNAeasy Microkit (Qiagen) supplemented with 1% β-mercaptoethanol (v/v), keeping a ratio of 3 volumes of RLT buffer per 1 volume of sorted cells (in our sorting conditions, 50,000 cells in 200 *µ*L). Cell sorting was performed using a Moflo XDP (Beckman Coulter Inc.). Each experiment yielded between 80,000 and 100,000 STK-GFP-positive cells.

### RNA extraction and expression analysis

RNA was extracted immediately after sorting using an RNAeasy Microkit (Qiagen) with a few modifications. We used one column for every 25,000 cells. Columns were eluted twice, using the first eluate for the second elution. Finally, all eluates from the same sample were pooled, yielding 70 to 100 ng of total RNA. For RT-qPCR validation, the columns were eluted a third time, using 14 *µ*L of nuclease-free water that was sequentially used to elute all the columns corresponding to the same sample. This yielded around 5 ng of total RNA that was retro-transcribed using the SuperScript III First-Strand Synthesis System (Invitrogen/Life Technologies). Then, the expression of *STK-GFP* was measured by RT-qPCR using a Thermal Cycler from Applied Biosystems and a QuantiTect SYBR Green PCR Kit (Qiagen). *MON1* (*AT2G28390*) was used as a reference gene for normalization (Czechowski et al. 2005).

For evaluation of *STM* levels in RNAi lines, RNA was extracted using Trizol (Invitrogen), and 500 ng of RNA was retrotranscribed using the iScript cDNA synthesis kit (BioRad). RT-qPCRs were performed using iTaq Universal SYBR Green Supermix (BioRad) in a Bio-Rad iCycler iQ thermal cycler. The *ACT8* gene (*AT1G49240*) was used as the reference gene for normalization because, according to our RNA-seq data, its RNA levels did not change in *hda19-3* plants.

All primers used in RT-qPCR experiments showed an amplification efficiency close to 100%, so the 2^−ΔΔCT^ method was used for the calculation of expression changes. All primers used are listed in Supplemental Table S1.

### RNA sequencing and analysis

Three samples of RNA from wild-type GFP-positive cells and four samples from *hda19-3* GFP-positive cells were used for library preparation. Libraries were sequenced in one lane of the HiSeq2500 Illumina platform with a yield of 250 million reads and an average of 35 million reads per sample. Around 10 million reads were filtered out. Of the 240 million reads remaining, 230 million were mapped uniquely (96% of the reads after filtering) and were used for analysis.

Library preparation, RNA sequencing, and bioinformatic analysis were essentially performed as described (Villarino et al. 2016). Briefly, we used Cufflinks (Trapnell et al. 2012), DeSeq2 (Love et al. 2014), and EdgeR (Robinson et al. 2009) to analyze the RNA-seq data (Supplemental Data Set 1 to S3). Then, we crossed the data obtained by the three software packages and selected the DEGs detected by all three programs, with an FDR < 0.001 and a logFC > |1.2| (Supplemental Data Set 4). Genes whose expression value was zero in either wild-type or *hda19-3* samples were filtered out.

### Chromatin immunoprecipitation and qPCR analysis

Chromatin immunoprecipitation was performed as described (Mizzotti et al. 2014). We used a rabbit anti-H3K9/K14ac antibody (Upstate 07-352, Sigma-Aldrich) for the immunoprecipitation of DNA fragments associated with acetylated histones and a mouse monoclonal anti-GFP antibody (Roche, #11814460001) for the immunoprecipitation of DNA fragments associated with STK-GFP. We collected the whole inflorescences, leaving the IM out by cutting the flowers (up to stage 12) just below their base with tweezers. qPCR was performed using iTaq Universal SYBR Green Supermix (BioRad) in a Bio-Rad iCycler iQ thermal cycler.

Enrichment of tested areas was calculated as fold-change against a control, nonenriched region (*GAPDH* locus) as previously described (Matias-Hernandez et al. 2010). Primers used for ChIP-qPCR are listed in Supplemental Table S1. For the anti-H3K9/K14ac antibody ChIP-qPCR experiment, statistical significance was evaluated by Student's *t*-test (two-tailed distribution, homoscedastic) comparing wild type with *hda19-3*, *stk* or *stkshp1shp2* among three independent biological replicates. For the anti-GFP antibody ChIP-qPCR, wild type and *pSTK::STK-GFP* enrichment values were compared with Student's *t*-test (two-tailed distribution, homoscedastic) among four independent biological replicates.

### In situ hybridization

Sectioning and ISH were performed as described (Dreni et al. 2007). The *STM* probe was obtained as described (Simonini and Kater 2014).

### Generation of RNAi lines

For the *p35S::STM-RNAi* construct, a fragment of 352 bp of *STM* coding sequence was amplified (see primer list in Supplemental Table S3), cloned into pDONR207, and then subcloned into pFRH (derived from pFGC5941; NCBI accession number AY310901) using Gateway technology (Invitrogen).

The construct was transformed into *Agrobacterium tumefaciens* strain EHA105, and *hda19-3* plants were transformed by the floral dip method (Clough and Bent 1998). Transformants were selected on MS media (Murashige and Skoog 1962) supplemented with 20 mg/L of hygromycin. The presence of the construct was confirmed by genotyping (see primers in Supplemental Table S1).

### Clustering analysis

Heatmaps were obtained using Heatmapper (Babicki et al. 2016). Genes were hierarchically clustered by Euclidean distances using the average linkage method.

### Accession numbers

Sequence data from this article can be found in the GenBank/EMBL data libraries under accession number PRJNA1031145.

Accession numbers of the major Arabidopsis genes mentioned in this work are as follows: *HDA19 =* AT4G38130; *STM =* AT1G62360; *STK =* AT4G09960; *SHP1* = AT3G58780; *SHP2* = AT2G42830.

## Supplementary Material

kiad629_Supplementary_Data

## Data Availability

RNA-seq data are available under the accession number PRJNA1031145 (www.ncbi.nlm.nih.gov/bioproject/PRJNA1031145).

## Supplemental data

The following materials are available in the online version of this article.

**Supplemental Figure S1.** Growth of pollen tubes in pistils of wild-type, *HDA19*/*hda19-3*, and *hda19-3* plants.

**Supplemental Figure S2.** STK-GFP expression in the *pSTK::STK-GFP* marker line.

**Supplemental Figure S3.** Enrichment of the *STK-GFP* transcript.

**Supplemental Figure S4.** Expression levels of histone acetylases and deacetylases in STK-GFP expressing *hda19-3* cells as compared to the wild type in RNA-seq data.

**Supplemental Figure S5.** Transmitting tract phenotypes of wild-type, *had19-3*, and *hda19-3 STMRNAi* plants.

**Supplemental Table S1.** Primers used in this study.

**Supplemental Data Set 1.** Results of RNA-seq analysis with Cufflinks.

**Supplemental Data Set 2.** Results of RNA-seq analysis with DeSeq2.

**Supplemental Data Set 3.** Results of RNA-seq analysis with EdgeR.

**Supplemental Data Set 4.** Common DEGs among Cufflinks, DeSeq2, and EdgeR.

**Supplemental Data Set 5.** Enrichment analysis of “Biological Process’ GO terms associated with the upregulated genes.

**Supplemental Data Set 6.** Enrichment analysis of “Biological Process’ GO terms associated with downregulated genes.

## Dive Curated Terms

The following phenotypic, genotypic, and functional terms are of significance to the work described in this paper:

BZR1 Gramene: AT1G75080
BZR1 Araport: AT1G75080

HDA6 Gramene: AT5G63110
HDA6 Araport: AT5G63110

HDA9 Gramene: AT3G44680
HDA9 Araport: AT3G44680

KNAT6 Gramene: AT1G23380
KNAT6 Araport: AT1G23380

MYB55 Gramene: AT4G01680
MYB55 Araport: AT4G01680

PIN1 Gramene: AT1G73590
PIN1 Araport: AT1G73590

HEC1 Gramene: AT5G67060
HEC1 Araport: AT5G67060

STM Gramene: At1g62360
STM Araport: At1g62360

ACT8 Gramene: AT1G49240
ACT8 Araport: AT1G49240

ARF4 Gramene: AT5G60450
ARF4 Araport: AT5G60450

SHP2 Gramene: AT2G42830
SHP2 Araport: AT2G42830

IAA18 Gramene: AT1G51950
IAA18 Araport: AT1G51950

HEC2 Gramene: AT3G50330
HEC2 Araport: AT3G50330

HEC3 Gramene: AT5G09750
HEC3 Araport: AT5G09750

STK Gramene: AT4G09960
STK Araport: AT4G09960

pollen AmiGo: PO:0025281

HDA19 Gramene: AT4G38130
HDA19 Araport: AT4G38130

SHP1 Gramene: AT3G58780
SHP1 Araport: AT3G58780

ovule primordium AmiGo: PO:0000018

gynoecium AmiGo: PO:0009062

pistil length Planteome: TO:0002610

meristematic cell AmiGo: PO:0004010

histone deacetylation Gramene: GO:0016575
histone deacetylation Araport: GO:0016575

ARF3 Gramene: AT2G33860
ARF3 Araport: AT2G33860

BP Gramene: AT4G08150
BP Araport: AT4G08150

CLV1 Gramene: AT1G75820
CLV1 Araport: AT1G75820

ANT Gramene: AT4G37750
ANT Araport: AT4G37750

NTT Gramene: AT3G57670
NTT Araport: AT3G57670

plant ovary placenta AmiGo: PO:0020001

pollen tube growth Gramene: GO:0009860
pollen tube growth Araport: GO:0009860

gynoecium development Gramene: GO:0048467
gynoecium development Araport: GO:0048467

plant ovule AmiGo: PO:0020003

FIL Gramene: AT2G45190
FIL Araport: AT2G45190

PHB Gramene: AT2G34710
PHB Araport: AT2G34710

STY1 Gramene: AT3G51060
STY1 Araport: AT3G51060

SPT Gramene: AT4G36930
SPT Araport: AT4G36930

YAB3 Gramene: AT4G00180
YAB3 Araport: AT4G00180

NGA4 Gramene: AT4G01500
NGA4 Araport: AT4G01500

PAN Gramene: AT1G68640
PAN Araport: AT1G68640

CUC1 Gramene: AT3G15170
CUC1 Araport: AT3G15170

CUC2 Gramene: AT5G53950
CUC2 Araport: AT5G53950
