# Supplementary material for: HISTONE DEACETYLASE19 Controls Ovule Number Determination and Transmitting Tract Differentiation
Source: Plant Physiol. 2023 Dec 7;194(4):2117–35. doi: 10.1093/plphys/kiad629 (PMC10980524; doi:10.1093/plphys/kiad629)
Supplement: kiad629_Supplementary_Data [file kiad629_supplementary_data.zip › Supplemental Data.pdf]

Supplemental Data for:

HISTONE DEACETYLASE19 CONTROLS OVULE NUMBER DETERMINATION AND TRANSMITTING TRACT DIFFERENTIATION

Silvia Manrique[1,†], Alex Cavalleri[1,†], Andrea Guazzotti[1,†], Gonzalo H Villarino[2], Sara Simonini[3], Aureliano Bombarely[1], Tetsuya Higashiyama[4,8], Ueli Grossniklaus[3], Chiara Mizzotti[1], Ana Marta Pereira[5,6], Silvia Coimbra[5,6], Subramanian Sankaranarayanan[7], Elisabetta Onelli[1], Simona Masiero[1], Robert G Franks[2], Lucia Colombo[1,*]

[1] Dipartimento di Bioscienze, Università degli Studi di Milano, Via Giovanni Celoria 26, 20133, Milan, Italy

[2] North Carolina State University, Department of Plant and Microbial Biology, Raleigh, NC, 27606, USA

[3] Department of Plant and Microbial Biology & Zurich-Basel Plant Science Center, University of Zurich, Zollikerstrasse 107, CH-8008, Zurich, Switzerland

[4] Institute of Transformative Bio-Molecules (ITbM), Nagoya University, Furo-cho, Chikusa-ku, Nagoya, Aichi 464-8601, Japan

[5] Faculdade de Ciências da Universidade do Porto, Departamento de Biologia, Universidade do Porto, rua do Campo Alegre, 4169-007 Porto, Portugal

[6] LAQV Requimte, Sustainable Chemistry, Universidade do Porto, 4169-007 Porto, Portugal

[7] Department of Biological Sciences and Engineering, Indian Institute of Technology Gandhinagar, Palaj, Gujarat 382355, India

[8] Present address: Department of Biological Sciences, Graduate School of Science, University of Tokyo, Yayoi 2-11-16, Bunkyo-ku, 113-0032 Tokyo, Japan

* Corresponding author: Colombo L. (lucia.colombo@unimi.it; L.C.)

[†]These authors contributed equally.

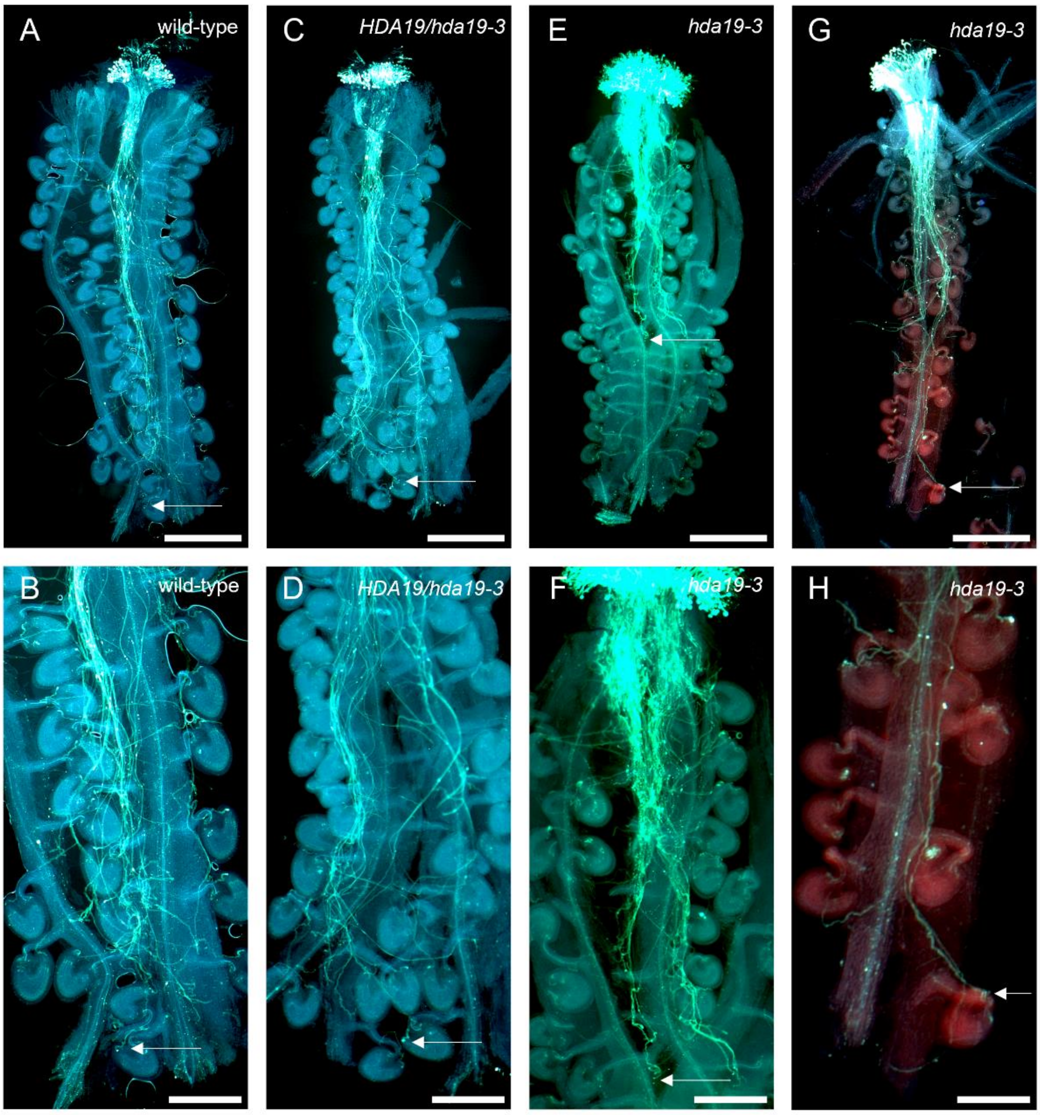

Supplemental Figure S1. Growth of pollen tubes in pistils of wild-type, *HDA19/hda19-3*, and *hda19-3* plants. A-B) Pollen tubes in a wild-type pistil at 12 HAP. **C-D)** Pollen tubes in an *hda19-3* heterozygous pistil at 12 HAP. **E-F)** Pollen tubes in an *hda19-3* homozygous pistil at 12 HAP. **G-H)** Pollen tubes in an *hda19-3* homozygous pistil at 24 HAP. Arrowheads mark the tip of pollen tubes. Images **B**, **D**, **F** and **H** (Scale bars = 50 µm) are enlargements of the portion of the pistil containing the longest pollen tubes of images **A**, **C**, **E** and **G** respectively (Scale bars = 100 µm).

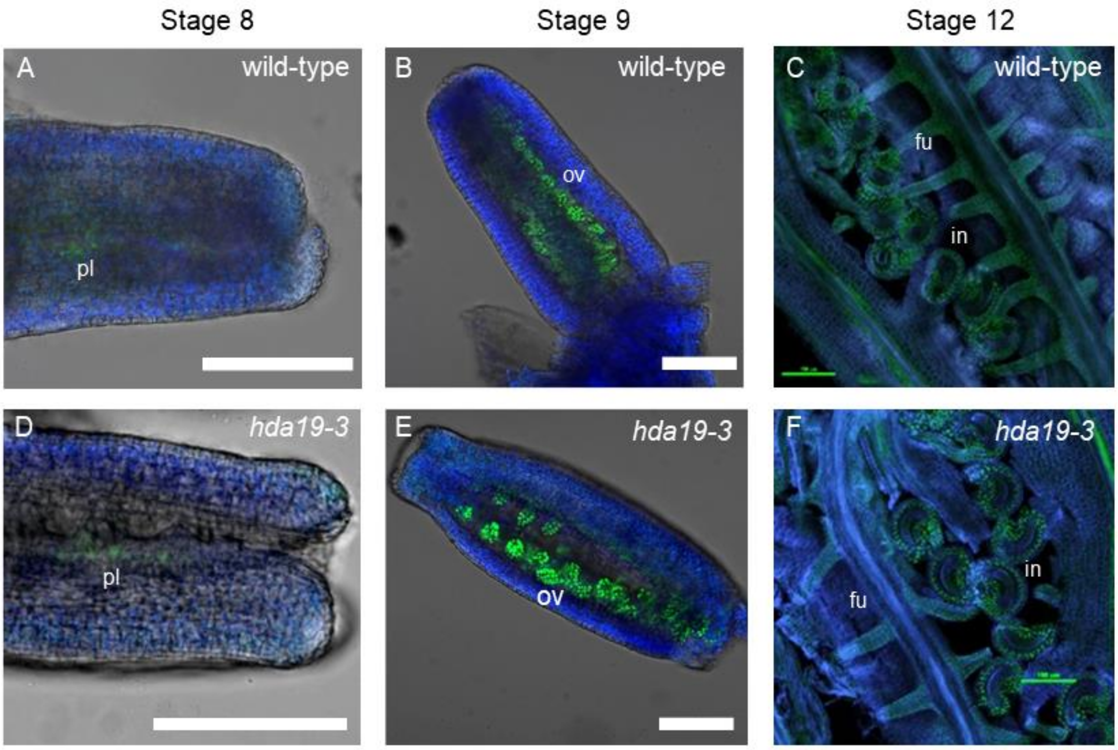

Supplemental Figure S2. STK-GFP expression in the *pSTK::STK-GFP* marker line. A-C) wild-type pistils at stages 8 (**A**), 9 (**B**) and 11 (**C**). **D-F)** *hda19-3* pistils at stages 8 (**D**), 9 (**E**) and 11 (**F**). The spatial expression pattern of the *pSTK::STK-GFP* marker line is maintained in both genotypes. First hints of expression are observed at stage 8 in the flat placenta (**A**, **D**). At stage 9, a strong expression in the ovule primordia and in the placenta can be observed, with weaker expression in the transmitting tract (**B**,**E**). At stage 11, fluorescence appears in the ovule integuments, funiculus, and placenta (**C**, **F**). Images were obtained with an A1 Nikon confocal microscope. Abbreviations: pl=placenta; OV= ovule; fu= funiculus; in= integuments. (**A**, **B**, **D**, **E**) scale bar = 50 µm. (**C**, **F**) scale bar = 100 µm.

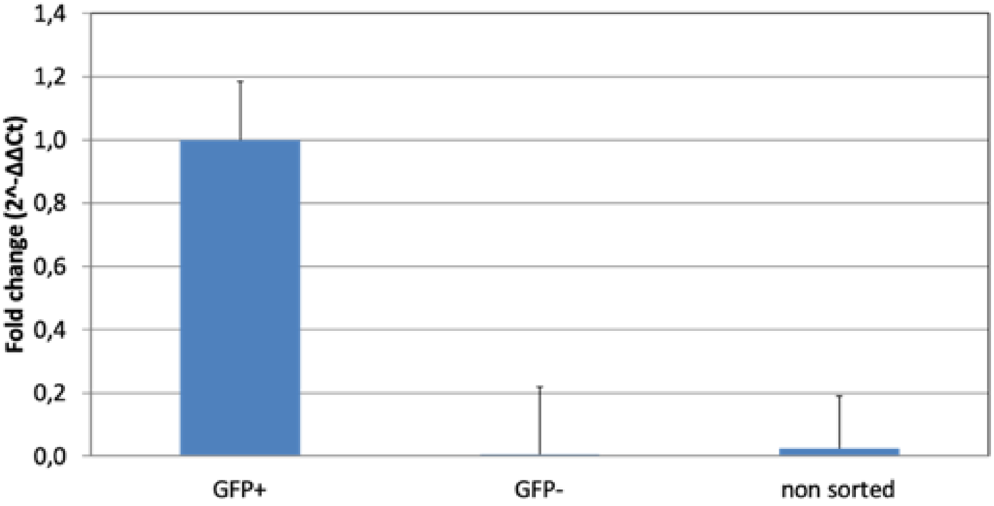

Supplemental Figure S3. Enrichment of *STK-GFP* transcript. Enrichment of *STK-GFP* transcript in cells sorted as GFP+, GFP- and non-sorted. *MON1* (*AT2G28390*) was used as reference gene for normalization. Error Bars represent the SE among three replicates.

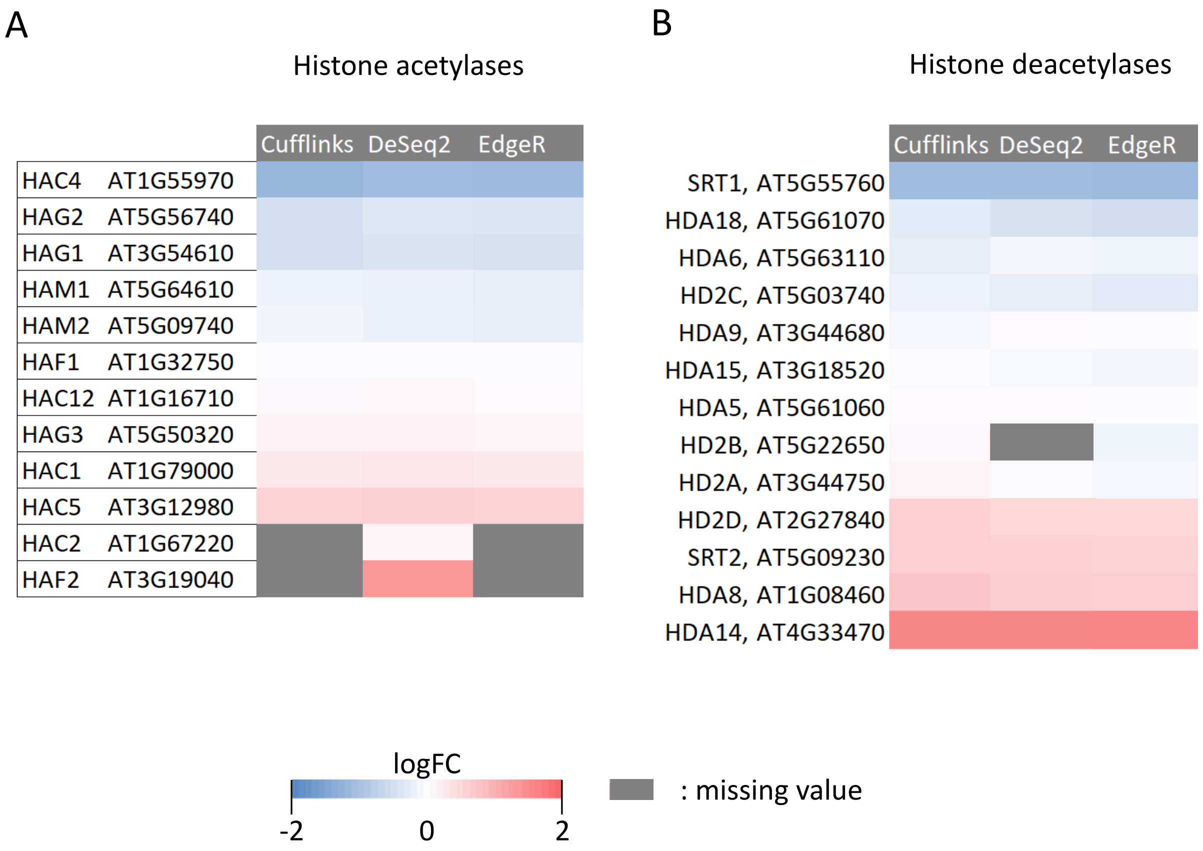

Supplemental Figure S4. Expression levels of histone acetylases and deacetylases in STK-GFP expressing *hda19-3* cells as compared to the wild type in RNA-seq data. A) Expression of histone acetylases in *hda19-3 pSTK::STK-GFP* expressing cells according to Cufflinks, DeSeq2 and EdgeR. **B)** Expression of histone deacetylases in *hda19-3 pSTK::STK-GFP* expressing cells according to Cufflinks, DeSeq2 and EdgeR. **A**, **B)** Color of cells represents the fold change (logFC) of each gene according to Cufflinks, DeSeq2 or EdgeR. Grey squares represent absence of logFC value due to inconsistent data according to the statistical parameters of the program. logFC and significance was calculated using the same parameters used for the analysis of the RNAseq mentioned in Figure 2 and in the Methods section.

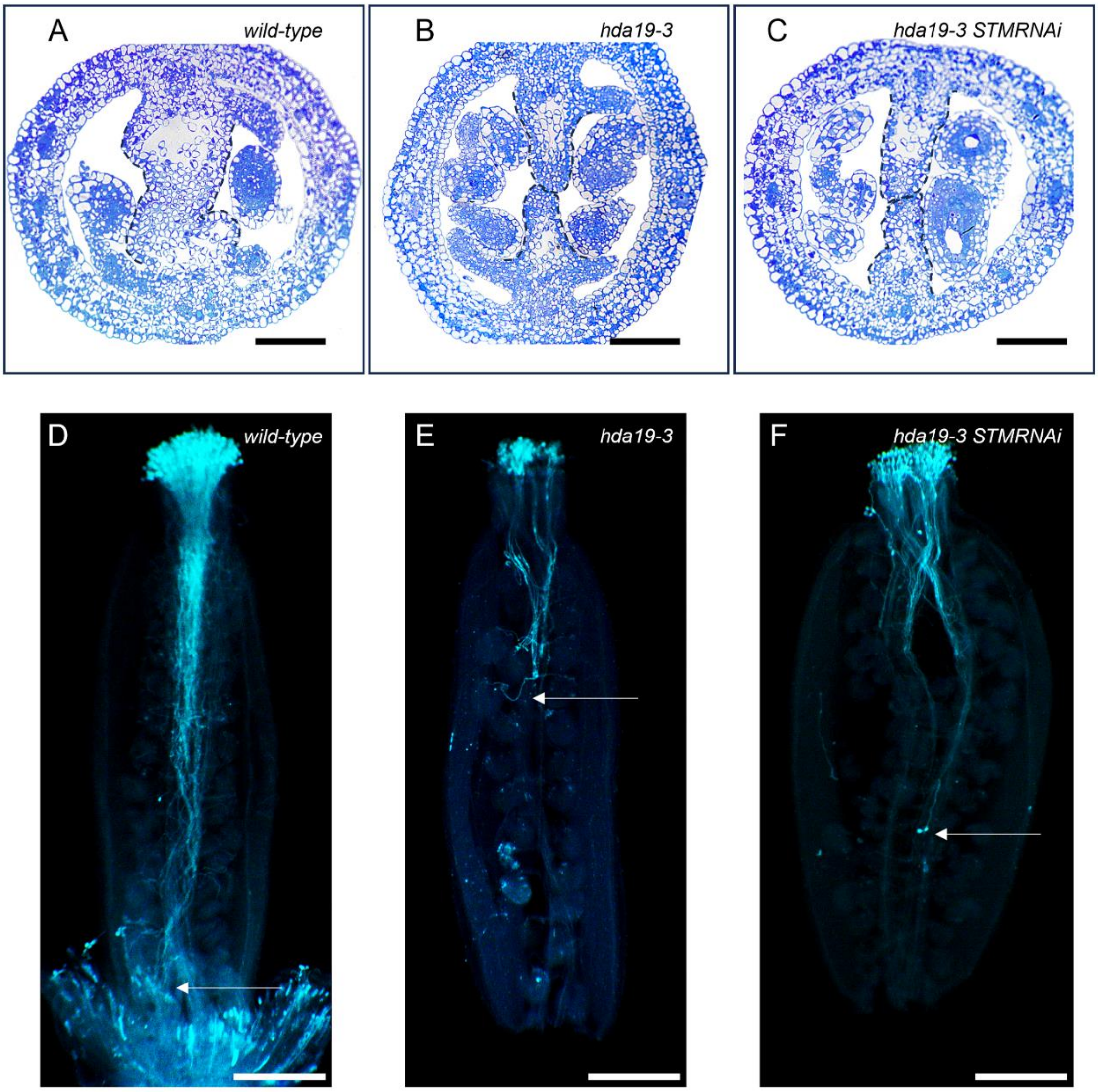

Supplemental Figure S5. Transmitting tract phenotypes of wild-type, *had19-3*, and *hda19-3 STMRNAi* plants. A-C) Transversal sections of wild-type (**A**), *hda19-3* (**B**) and an example of an *hda19-3 STMRNAi* line (**C**). Images were digitally extracted for comparison. Scale bars = 50 µm. **D-F)** Aniline blue staining of pollen tubes at 14 HAP in wild-type (**D**), *hda19-3* (**E**) and one example of *hda19-3 STMRNAi* (**F**). White arrowheads indicate the position of pollen tubes. Scale bars = 100 µm.

Supplemental Table S1. Primers used in this study

Primer	Sequence	Usage
STK-GFP FWD	TTGAGCTTGACAATGAGAACATC	Expression analysis of *STK* gene (validation of sorted RNA samples)
STK-GFP REV	GGTTTTTCGCAGACGAGATT	
MON1 FWD	CAGACAAGGCGATGGCGATA	Expression analysis of *MON1* gene
MON1 REV	GCTTTCTCTCAAGGGTTTCTGGGT	
ACT 8 FWD	CTCAGGTATTGCAGACCGTATGAG	Expression analysis of *ACT8* gene
ACT8 REV	CTGGACCTGCTTCATCATACTCTG	
STM ISH FWD	GTTGCTTCTTCTTCTTCTCC	Synthesis of STM probe for *in situ* hybridisatio n
STM ISH REV + T7	TAATACGACTCACTATAGGGACGAGCATT TCACAGTAAGC	
STM_FWD	CCTTCAACGTGTCGAGTGTC	Expression analysis of *STM* gene
STM_REV	ACTTCTTCCTCGGATGACCC	
STM RNAI FWD	GGGGACAAGTTTGTACAAAAAAGCAGGC TAACCCTTGCTCCTCTTCCTC	Gateway cloning of a fragment of *STM* CDS for RNAi construct
STM RNAI REV	GGGGACCACTTTGTACAAGAAAGCTGGG TACCGGAGAAAGAGGAAGGTG	
STM_RNAI_G TPYING FWD	CCTCTGTCAAGGCCAAGATC	Genotyping of STM-RNAi plants (construct is distinguishe d from endogenous gene because primers span two exons)
STM_RNAI_G TPYING REV	GACACTCGACACGTTGAAGG	
STM_H3K9_C HIP_FWD	ACTTTGTTGGTGGTGTGACTG	*STM* TSS region
STM_H3K9_C HIP_REV	ATGATGATGATGATGCCGCC	

STM_H3K9_CHIP_FWD	TCTATGAGCGTAGGAGAC	*STM* -2Kb region
STM_H3K9_CHIP_REV	CCAAAATATGTTGGATCTGGAC	
STM_CARG_I1_FWD	TGTTTACTAGTTACTTAACCCAGCT	*STM* first intron CARG-BOX
STM_CARG_I1_REV	TGGATAATCTCTTGCAAGTAGGGT	
STM_CARG_I2_FWD	TGCTCGTCCTTAGATCTATTGCT	*STM* second intron CARG-BOX
STM_CARG_I2_REV	AGTAGATGTGAGTTTGTGTGTCT	
STM_CARG_PROM_FWD	TTCACTGGACTTTCCGAGGC	*STM* promoter CARG-BOX
STM_CARG_PROM_REV	TGATTTCTCATTTATGCCTTTTCGGA	
VDD FWD	GGAAATATGACGCTTGTCTTTTTAG	Positive control STK-GFP CHIP
VDD REV	CAGAAACAGCAATATGCTCGTG	
GAPDH FWD	CTCGTTGTGCAGGTCTCAAA	Normalizer for CHIP experiments
GAPDH REV	CTAGTGGCTCATCGCAGA	
