# Supplementary material for: HISTONE DEACETYLASE19 Controls Ovule Number Determination and Transmitting Tract Differentiation
Source: Plant Physiol. 2023 Dec 7;194(4):2117–35. doi: 10.1093/plphys/kiad629 (PMC10980524; doi:10.1093/plphys/kiad629)
Supplement: kiad629_Supplementary_Data [file PP2023RA01337DR1Merged_Decision_Summary_PDF.pdf › PP2023RA01337DR1Merged_Decision_Summary_PDF.pdf]

Plant Physiology®

HDA19 CONTROLS OVULE NUMBER DETERMINATION AND TRANSMITTING TRACT DIFFERENTIATION BY REGULATING CMM ACTIVITY

Silvia Manrique, Alex Cavalleri, Andrea Guazzotti, Gonzalo Villarino, Sara Simonini, Aureliano Bombarely, Tetsuya Higashiyama, Ueli Grossniklaus, Chiara Mizzotti, Ana Pereira, Silvia Coimbra, Subramanian Sankaranarayanan, Elisabetta Onelli, Simona Masiero, Robert Franks, and Lucia Colombo

Review Timeline:

Submission Date:	25-May-2023
Editorial Decision:	30-Jul-2023
Resubmission Received:	30-Aug-2023
Editorial Decision:	02-Oct-2023
Revision Received:	27-Oct-2023
Accepted:	29-Oct-2023

30-Jul-2023

Prof. Lucia Colombo
Universita degli Studi di Milano
Biosciences
Milano 20133
Italy

RE: HDA19 CONTROLS OVULE NUMBER DETERMINATION AND TRANSMITTING TRACT DIFFERENTIATION BY REGULATING THE CMM ACTIVITY

Dear Lucia:

First of all, I would like to apologize that it took quite long to evaluate the manuscript, but it was quite difficult to find the minimum of requested reviewers.

As you can read below, the two reviewers have found the manuscript of interest, but have raised a number of concerns that will require your attention. As these concerns are of some gravity and will entail substantial changes to the manuscript, you are likely to need additional time to address them. I must therefore reject the manuscript in its present form. Nonetheless, if you are able to address the points raised in review, I would welcome your resubmission of a manuscript that incorporates your responses.

In particular, I direct your attention to the following comments for consideration in a resubmitted manuscript.

Overall, while comments of Reviewer #1 highlights positive aspects of the manuscript and provides a number of constructive suggestions for improvement, Reviewer #2's comments are more critical; it is suggested that a larger number of important issues have to be addressed to strengthen the study. Some results and figures appear preliminary and the whole manuscript should be better organized as outlined below. A number of questions have been addressed that need clarification. My major concern is, however, that the main conclusion that epigenetic regulation by HDA19 controls ovule initiation and transmitting tract differentiation via affecting the acetylation level appears not fully justified as acetylation levels were not investigated.

Please note that any resubmission will be treated as a new entry to the system and is likely to go out for review.

If you do have any queries or concerns, you are welcome to contact me in the first instance.

Thank you again for allowing Plant Physiology to review your work. We look forward to hearing from you soon.

Sincerely,

Plant Physiology Board of Editors

-- Reviewer comments:
Reviewer #1 (Comments for the Author):

The manuscript entitled "HDA19 controls ovule number determination and transmitting tract differentiation by regulating the CMM activity" reveals the function of the HDA19 gene to downregulate SHOOT MERISTEMLESS (STM) in the carpel margin meristem (CMM), allowing ovule formation and transmitting tract (TT) differentiation. Overall, the manuscript provides valuable insights into the molecular mechanisms underlying ovule development and the regulatory roles of HDA19. The study addresses an important gap in understanding the genetic and molecular regulation of ovule number and transmitting tract development. The findings shed light on the roles of HDA19 in controlling ovule initiation and tissue differentiation, and their interaction in regulating gene expression patterns. The manuscript is well-organized, with a clear introduction, methods, results, and discussion sections. The figures and supplementary data provided support the findings and aid in the interpretation of the results. The logical flow of the manuscript enhances the readability and comprehension of the study.
Suggestions for improvement:
1. Although the authors demonstrate the upregulation of STM and other genes in hda19-3 mutant and the rescue of ovule defects by reducing STM levels, however, it would be valuable to include additional functional evidence, such as complementation assays with other known regulators or downstream targets of HDA19 or STK, to further support their conclusions.

2. Are there other factors or regulators that might contribute to the phenotypes observed in hda19-3 mutant?
3. Consider rephrasing sentences 66-69 to improve clarity. The current phrasing may cause confusion regarding the relationship between ovule fertilization, seed formation, and the emergence of the reproductive tract (ReT) and ovules from the carpel margin meristem (CMM).
4. In the discussion the authors could provide a more detailed and mechanistic discussion of the proposed interaction between HDA19 and STK, such as potential post-translational modifications or indirect regulation.
5. The detail about the sample and stages of the flower used for the ChIP-qPCR should be included.

Reviewer #2 (Comments for the Author):

Comments:
The manuscript entitled "HDA19 CONTROLS OVULE NUMBER DETERMINATION AND RANSMITTING TRACT DIFFERENTIATION BY REGULATING THE CMM 2 ACTIVITY" studied HDA19 role in ovule number determination and ReT differentiation. hda19-3 mutant displays reduced ovule number and fails to properly differentiate the TT. The author used FACS coupled with RNA-seq to reveal that genes promoting organ development are downregulated in the CMM of hda19-3 mutant while meristematic markers (including STM) are upregulated, which means HDA19 downregulates STM in the CMM, allowing ovule formation and TT differentiation. STM is ectopically expressed in hda19-3 at intermediate stages of pistil development, and its downregulation by RNA interference alleviated hda19-3 phenotypic defects. ChIP indicated that STM is a target of HDA19 during pistil development and that STK is required for the histone acetylation-mediated regulation of STM. Basically, I considered that this work is important and interesting. The epigenetic regulation of ovule initiation is a good point. However, it seemed that the authors are too hurry to submit this manuscript. Many results and figures are too preliminary and the organization of this manuscript is not logically enough. The conclusions didn't include some important phenotypes and mechanisms, or, solve some essential questions. I suggested that this study could not been considered to publish in Plant Physiology until the authors should reorganize the manuscript and carefully address all the questions raised by reviewers and editor. The main issues are followed:

1. HDA19, HDA6, and other HISTONE DEACETYLASEs are reported to involved in the growth and development of plants, such as flowering, leaf formation, and embryonic development. However, the author directly selected HDA19 as the aim gene from the very beginning of this study, and only the HDA19 mutant was used for analysis. How about other HDAs? Do they also have similar phenotypes during ovule number determination and TT differentiation? Are their deacetylation functions redundant during the development of pistils and ovules? The author should provide the reason for studying HDA19 in these processes.
2. In the analysis of FACS coupled with RNA-seq, there are several upregulated genes were identified which supposed to be related to TT development. Why did the author only select STM for subsequent research (line 279) but not others, such as CLV1? In addition, the hda19-3 STM RNAi line 1 and hda19-3 STM RNAi line 10 selected by the authors were not the lines with lowest STM transcription levels, but their complementary efficiencies of TT were the best among all lines. Is it possibly indicated that there is no direct relationship between STM transcription levels and TT development?
3. HDA19 does not directly bind to DNA, therefore it must interact with transcription factors or other cofactors to regulate its target. Before flower developmental stage 8, there are many transcription factors that can be candidates. Why did the authors directly use STK (line 370) as the only candidate and conduct genetic experiments?
4. The most important point of this manuscript is the epigenetic regulation by HDA19 during ovule initiation and TT development. Since ChIP assay only reveals the binding of protein to DNA but not the acetylation, the conclusion of acetylation level is a little bit overstate. It supposed to be direct assay to detect the acetylation in hda19 mutant and WT.
5. Previous studies have shown that HDA19 interacts with ETT, ARF4, and FIL to regulate STM in IM (Chung et al., 2019), indicating that the function of HDA19 has been reported and HDA19 affects different processes of growth and development. HDA19 mutant has many abnormal phenotypes in the process of pistil development and double fertilization, such as influencing the initial number of ovules without affecting the pistil length. Why did the authors only study TT in this work (the authors mentioned ovule number determination in the title but focus on TT development in the manuscript). Besides, the author's description of TT abnormalities was not clear enough. What is the conclusion, the ectopic proliferation of cells caused by STM overexpression (Figure 1 E-H, Figure 4 F-G), or the abnormal phenotype of TT caused by non-fusion septum (Figure S5 A-C), and are both related to the function of STM? The authors tried to include many points in one paper, which leads to unclear conclusion in each point.
6. This study focuses on the phenotype of TT by using pSTK: STK-GFP mediated FACS for RNA-seq. It is known that STK is expressed in the placenta before ovule initiation and in the ovules lately. Is STK expressed in TT? Figure S2 did not capture the STK fluorescence at TT, please double check it.
7. Because the FACs coupled RNA-seq is a very precise experiment, the authors should describe the methods very clearly. In this article, the developmental stage of flowers collected for materials are very important. The authors described that they collected flowers at stages 1-12. However, according to experiences, the pistils of the 1-6 stages are very difficult to separate (barely to see). How did the author do that, directly take the entire inflorescence apex, or collected buds then remove other parts to leave the pistils? There is no detailed description in the materials and methods. Please provide details in the section of sampling process.
8. The figures are not finalized. The background of plants and the processing time are not labeled in almost all images. For

example, in Figure 1, there are different developmental stage of ovules in the same placenta at flower developmental stage 9, which are not reflected in the model of ovule development. Are Figure 1B, C, E, F, G, H mutant or WT? As well as Figures 4 A-H, 6 A-H, S1, S2, etc. The readers only can find the information from brief legend. In addition, the quality of many images in this article needs to be improved. The current images seemed not to be good enough and insufficient to gain conclusions. Please replace them with high-quality images. For example, the staining assay of pollen tube in Figure S1. Figure G-H showed an incomplete pistil, with the bottom cut off. The fluorescence intensity and white balance of each piece of image are different. The ovules on all placentae crowded and couldn't be seen clearly, suggesting the results and figures are preliminary.

Outline of author's responses to editor's and reviewer's comments (authors responses written in bold):

Editor comment:
"My major concern is, however, that the main conclusion that epigenetic regulation by HDA19 controls ovule initiation and transmitting tract differentiation via affecting the acetylation level appears not fully justified as acetylation levels were not investigated".

Acetylation ChIP of STM is indeed present in the manuscript. The acetylation level of *STM* in *hda19* with respect to the wild type has been analyzed (see Figure 4I-J). Acetylation levels in stk mutant were also analyzed (Figure 7A). We have added a sentence to emphasize the nature (lines 289-291 and 382-395) and importance (lines 302-305, 471-474) of these experiments.

-- Reviewer comments:
Reviewer #1 (Comments for the Author):

The manuscript entitled "HDA19 controls ovule number determination and transmitting tract differentiation by regulating the CMM activity" reveals the function of the HDA19 gene to downregulate SHOOT MERISTEMLESS (STM) in the carpel margin meristem (CMM), allowing ovule formation and transmitting tract (TT) differentiation. Overall, the manuscript provides valuable insights into the molecular mechanisms underlying ovule development and the regulatory roles of HDA19. The study addresses an important gap in understanding the genetic and molecular regulation of ovule number and transmitting tract development. The findings shed light on the roles of HDA19 in controlling ovule initiation and tissue differentiation, and their interaction in regulating gene expression patterns. The manuscript is well-organized, with a clear introduction, methods, results, and discussion sections. The figures and supplementary data provided support the findings and aid in the interpretation of the results. The logical flow of the manuscript enhances the readability and comprehension of the study. Suggestions for improvement:

1. Although the authors demonstrate the upregulation of STM and other genes in hda19-3 mutant and the rescue of ovule defects by reducing STM levels, however, it would be valuable to include additional functional evidence, such as complementation assays with other known regulators or downstream targets of HDA19 or STK, to further support their conclusions.

Our manuscript reports unique cell-specific transcriptome datasets to identify new possible master regulators that must be silenced by HDA19 for correct transmitting tract development and ovule number regulation. These datasets are of considerable value to those studying developmental processes related to plant reproduction. To further support the importance of our datasets, we focused on STM, one of the few master regulators that was up-regulated in the *hda19* mutant and whose up-regulation could explain the phenotype that we observed in the *hda19* mutant.

We agree with the reviewer that some other genes involved in transmitting tract development and ovule number regulation that we and other groups have already studied could be very interesting to include (Cucinotta et al., 2016; Cucinotta et al., 2018; Gomez et

al., 2018; Herrera-Ubaldo et al., 2019; DiMarzo et al., 2020). However, the genes known to be important for ovule number determination and TT differentiation are downregulated in HDA19, suggesting that they probably are not HDA19 direct targets.

2. Are there other factors or regulators that might contribute to the phenotypes observed in hda19-3 mutant?

Yes, we indeed think this is the case. We have added a paragraph discussing this topic. For more details, see lines 424-439 and 445-450.

3. Consider rephrasing sentences 66-69 to improve clarity. The current phrasing may cause confusion regarding the relationship between ovule fertilization, seed formation, and the emergence of the reproductive tract (ReT) and ovules from the carpel margin meristem (CMM).

We have changed the sentence as suggested (see lines 65-68)

4. In the discussion the authors could provide a more detailed and mechanistic discussion of the proposed interaction between HDA19 and STK, such as potential post-translational modifications or indirect regulation.

We have included, as suggested by reviewer 1, a more detailed description of possible modes of interaction between HDA19 and STK. See lines 497-517.

5. The detail about the sample and stages of the flower used for the ChIP-qPCR should be included.

We have included a more detailed description of the stages of the flower used for the experiments, as suggested. See lines 643–647.

Reviewer #2 (Comments for the Author)

Comments:

The manuscript entitled "HDA19 CONTROLS OVULE NUMBER DETERMINATION AND RANSMITTING TRACT DIFFERENTIATION BY REGULATING THE CMM 2 ACTIVITY" studied HDA19 role in ovule number determination and ReT differentiation. hda19-3 mutant displays reduced ovule number and fails to properly differentiate the TT. The author used FACS coupled with RNA-seq to reveal that genes promoting organ development are downregulated in the CMM of hda19-3 mutant while meristematic markers (including STM) are upregulated, which means HDA19 downregulates STM in the CMM, allowing ovule formation and TT differentiation. STM is ectopically expressed in hda19-3 at intermediate stages of pistil development, and its downregulation by RNA interference alleviated hda19-3 phenotypic defects. ChIP indicated that STM is a target of HDA19 during pistil development and that STK is required for the histone acetylation-mediated regulation of STM. Basically, I considered that this work is important and interesting. The epigenetic regulation of ovule initiation is a good point. However, it seemed that the authors are too hurry to submit this manuscript. Many results and figures are too preliminary, and the organization of this manuscript is not logically enough. The conclusions didn't include some important phenotypes and mechanisms, or, solve some essential questions. I suggested that this study could not been considered to publish in Plant Physiology until the authors should reorganize the manuscript and carefully address all the questions raised by reviewers and editor. The main issues are followed:

1. HDA19, HDA6, and other HISTONE DEACETYLASEs are reported to involved in the growth and development of plants, such as flowering, leaf formation, and embryonic development. However, the

author directly selected HDA19 as the aim gene from the very beginning of this study, and only the HDA19 mutant was used for analysis. How about other HDAs? Do they also have similar phenotypes during ovule number determination and TT differentiation? Are their deacetylation functions redundant during the development of pistils and ovules? The author should provide the reason for studying HDA19 in these processes.

We have included in the revised manuscript a more detailed description of the rationale driving our choice, focusing on HDA19 (lines 148–155).

2. In the analysis of FACS coupled with RNA-seq, there are several upregulated genes were identified which supposed to be related to TT development. Why did the author only select STM for subsequent research (line 279) but not others, such as CLV1?

We agree with reviewer 2 that this could be better explained, and we have added a more detailed description to explain the rationale of the choice that prompted us to prioritize STM over the other upregulated meristematic regulators (lines 269–275). We also discuss this point in more detail in the discussion (see lines 424-439).

In addition, the hda19-3 STM RNAi line 1 and hda19-3 STM RNAi line 10 selected by the authors were not the lines with lowest STM transcription levels, but their complementary efficiencies of TT were the best among all lines. Is it possibly indicated that there is no direct relationship between STM transcription levels and TT development?

We agree with Review 2 that a more detailed explanation is required, and we have added it to the revised manuscript. See lines 315–316 and 332-334.

3. HDA19 does not directly bind to DNA, therefore it must interact with transcription factors or other cofactors to regulate its target. Before flower developmental stage 8, there are many transcription factors that can be candidates. Why did the authors directly use STK (line 370) as the only candidate and conduct genetic experiments?

We have added a more detail explanation of the rationale of choosing STK (see lines 367-374).

4. The most important point of this manuscript is the epigenetic regulation by HDA19 during ovule initiation and TT development. Since ChIP assay only reveals the binding of protein to DNA but not the acetylation, the conclusion of acetylation level is a little bit overstate. It supposed to be direct assay to detect the acetylation in hda19 mutant and WT.

We believe this is a misunderstanding. As stated in lines 286-291 we performed a ChIP-qPCR experiment using an antibody against acetylated histones (acH3K9): "To analyse whether the changes in *STM* expression pattern could be caused by variations in its histone acetylation levels resulting from the absence of HDA19, we collected flowers from stages 1-12 (excluding the IM) from *wild-type* and *hda19-3* plants and performed ChIP-qPCR. We used an antibody against HISTONE 3 (H3) acetylated in Lysine 9 and 14 (K9/14) (AcH3K9/14) (Figure 4I-J) to capture DNA fragments associated with acetylated histones".

Nevertheless, we have explained these experiments also in the material and methods (see lines 643-647).

5. Previous studies have shown that HDA19 interacts with ETT, ARF4, and FIL to regulate STM in IM (Chung et al., 2019), indicating that the function of HDA19 has been reported and HDA19 affects different processes of growth and development. HDA19 mutant has many abnormal phenotypes in the

process of pistil development and double fertilization, such as influencing the initial number of ovules without affecting the pistil length. Why did the authors only study TT in this work (the authors mentioned ovule number determination in the title but focus on TT development in the manuscript).

We believe this is a misunderstanding, as s a significant part of the results is dedicated to the ovule number phenotype in the hda19 mutant (Figure 1B-D), in the complementing lines (Figure 5B-C), as well as that of the stk and stk shp1 shp2 mutants (Figure 6I-J).

Besides, the author's description of TT abnormalities was not clear enough. What is the conclusion, the ectopic proliferation of cells caused by STM overexpression (Figure 1 E-H, Figure 4 F-G), or the abnormal phenotype of TT caused by non-fusion septum (Figure S5 A-C), and are both related to the function of STM? The authors tried to include many points in one paper, which leads to unclear conclusion in each point.

We believe the reviewer mixed up ectopic expression with ectopic proliferation. What we have explained in our manuscript is that the ectopic expression of STM led to downregulation of genes involved in the differentiation process, as was shown for another meristem. In the text, 'ectopic proliferation' is not used, and it is unclear what the reviewer means by it.

6. This study focuses on the phenotype of TT by using pSTK: STK-GFP mediated FACS for RNA-seq. It is known that STK is expressed in the placenta before ovule initiation and in the ovules lately. Is STK expressed in TT? Figure S2 did not capture the STK fluorescence at TT, please double check it.

We have included a higher magnification of supplemental Figure S2. The objective of S2 is the comparison between STK::STK-GFP expression in wt with respect to the hda19 mutant. The STK::STK-GFP expression in wild-type TT and ovules was previously described in more than one manuscript, which we cited. Herrera-Ubaldo et al., 2019, Mizzotti et al., 2014, and Di Marzo et al., 2020.

7. Because the FACs coupled RNA-seq is a very precise experiment, the authors should describe the methods very clearly. In this article, the developmental stage of flowers collected for materials are very important. The authors described that they collected flowers at stages 1-12. However, according to experiences, the pistils of the 1-6 stages are very difficult to separate (barely to see). How did the author do that, directly take the entire inflorescence apex, or collected buds then remove other parts to leave the pistils?

It is not necessary to collect pistils using organ-specific cell sorting. We have added a more detailed description of the sampling and protoplast sorting process in the material and methods section (see lines 587–589).

There is no detailed description in the materials and methods. Please provide details in the section of sampling process. The figures are not finalized. The background of plants and the processing time are not labeled in almost all images. For example, in Figure 1, there are different developmental stage of ovules in the same placenta at flower developmental stage 9, which are not reflected in the model of ovule development. Are Figure 1B, C, E, F, G, H mutant or WT? As well as Figures 4 A-H, 6 A-H, S1, S2, etc. The readers only can find the information from brief legend.

Information regarding the different panels was available in the legend of the figures. We agree with reviewer 2 that this makes it necessary to read the legend to understand the figure, which might be inconvenient. We can add additional information to the panels (i.e., genotype, stage of development) to improve their readability.

In addition, the quality of many images in this article needs to be improved. The current images seemed not to be good enough and insufficient to gain conclusions. Please replace them with high-quality images. For example, the staining assay of pollen tube in Figure S1. Figure G-H showed an incomplete pistil, with the bottom cut off. The fluorescence intensity and white balance of each piece of image are different. The ovules on all placentae crowded and couldn't be seen clearly, suggesting the results and figures are preliminary.

The top panels of Supplemental Figure 1 (A, C, G, and H) show pistils where valves have been removed for clearer visualization of the growth of the pollen tubes. They are not cut, but as the valves have been removed, the bottom of the TT is straight, which might give the wrong impression that the pistil is cut. One can realize that this is not the case by checking the size of each pistil (they are all similar and the scale is the same) or by counting the ovules (the number of ovules present in each placenta is the typical number of the depicted genotype).Supplemental Figure 1 gives support to Figure 1I, where a graph showing the extent of pollen tube growth in the pistils of the different phenotypes was measured. The relevant information in this figure is the pollen tube growth, not the number of ovules.

Editor comment:

"My major concern is, however, that the main conclusion that epigenetic regulation by HDA19 controls ovule initiation and transmitting tract differentiation via affecting the acetylation level appears not fully justified as acetylation levels were not investigated".

Acetylation ChIP of STM is indeed present in the manuscript. The acetylation level of *STM* in *hda19* with respect to the wild type has been analyzed (see Figure 4I-J). Acetylation levels in stk mutant were also analyzed (Figure 7A). We have added a sentence to emphasize the nature (lines 289-291 and 382-395) and importance (lines 302-305, 471-474) of these experiments.

-- Reviewer comments:

Reviewer #1 (Comments for the Author):

The manuscript entitled "HDA19 controls ovule number determination and transmitting tract differentiation by regulating the CMM activity" reveals the function of the HDA19 gene to downregulate SHOOT MERISTEMLESS (STM) in the carpel margin meristem (CMM), allowing ovule formation and transmitting tract (TT) differentiation. Overall, the manuscript provides valuable insights into the molecular mechanisms underlying ovule development and the regulatory roles of HDA19. The study addresses an important gap in understanding the genetic and molecular regulation of ovule number and transmitting tract development. The findings shed light on the roles of HDA19 in controlling ovule initiation and tissue differentiation, and their interaction in regulating gene expression patterns. The manuscript is well-organized, with a clear introduction, methods, results, and discussion sections. The figures and supplementary data provided support the findings and aid in the interpretation of the results. The logical flow of the manuscript enhances the readability and comprehension of the study. Suggestions for improvement:

1. Although the authors demonstrate the upregulation of STM and other genes in hda19-3 mutant and the rescue of ovule defects by reducing STM levels, however, it would be valuable to include additional

functional evidence, such as complementation assays with other known regulators or downstream targets of HDA19 or STK, to further support their conclusions.

Our manuscript reports unique cell-specific transcriptome datasets to identify new possible master regulators that must be silenced by HDA19 for correct transmitting tract development and ovule number regulation. These datasets are of considerable value to those studying developmental processes related to plant reproduction. To further support the importance of our datasets, we focused on STM, one of the few master regulators that was up-regulated in the *hda19* mutant and whose up-regulation could explain the phenotype that we observed in the *hda19* mutant.

We agree with the reviewer that some other genes involved in transmitting tract development and ovule number regulation that we and other groups have already studied could be very interesting to include (Cucinotta et al., 2016; Cucinotta et al., 2018; Gomez et al., 2018; Herrera-Ubaldo et al., 2019; DiMarzo et al., 2020). However, the genes known to be important for ovule number determination and TT differentiation are downregulated in HDA19, suggesting that they probably are not HDA19 direct targets.

2. Are there other factors or regulators that might contribute to the phenotypes observed in hda19-3 mutant?

Yes, we indeed think this is the case. We have added a paragraph discussing this topic. For more details, see lines 424-439 and 445-450.

3. Consider rephrasing sentences 66-69 to improve clarity. The current phrasing may cause confusion regarding the relationship between ovule fertilization, seed formation, and the emergence of the reproductive tract (ReT) and ovules from the carpel margin meristem (CMM).

We have changed the sentence as suggested (see lines 65-68)

4. In the discussion the authors could provide a more detailed and mechanistic discussion of the proposed interaction between HDA19 and STK, such as potential post-translational modifications or indirect regulation.

We have included, as suggested by reviewer 1, a more detailed description of possible modes of interaction between HDA19 and STK. See lines 497-517.

5. The detail about the sample and stages of the flower used for the ChIP-qPCR should be included.

We have included a more detailed description of the stages of the flower used for the experiments, as suggested. See lines 643–647.

Reviewer #2 (Comments for the Author)

Comments:

The manuscript entitled "HDA19 CONTROLS OVULE NUMBER DETERMINATION AND RANSMITTING TRACT DIFFERENTIATION BY REGULATING THE CMM 2 ACTIVITY" studied HDA19 role in ovule number determination and ReT differentiation. hda19-3 mutant displays reduced ovule number and fails to properly differentiate the TT. The author used FACS coupled with RNA-seq to reveal that genes promoting organ development are downregulated in the CMM of hda19-3 mutant while meristematic markers (including STM) are upregulated, which means HDA19 downregulates STM in the CMM, allowing ovule formation and TT differentiation. STM is ectopically expressed in hda19-3 at intermediate

stages of pistil development, and its downregulation by RNA interference alleviated hda19-3 phenotypic defects. ChIP indicated that STM is a target of HDA19 during pistil development and that STK is required for the histone acetylation-mediated regulation of STM. Basically, I considered that this work is important and interesting. The epigenetic regulation of ovule initiation is a good point. However, it seemed that the authors are too hurry to submit this manuscript. Many results and figures are too preliminary, and the organization of this manuscript is not logically enough. The conclusions didn't include some important phenotypes and mechanisms, or, solve some essential questions. I suggested that this study could not been considered to publish in Plant Physiology until the authors should reorganize the manuscript and carefully address all the questions raised by reviewers and editor. The main issues are followed:

1. HDA19, HDA6, and other HISTONE DEACETYLASEs are reported to involved in the growth and development of plants, such as flowering, leaf formation, and embryonic development. However, the author directly selected HDA19 as the aim gene from the very beginning of this study, and only the HDA19 mutant was used for analysis. How about other HDAs? Do they also have similar phenotypes during ovule number determination and TT differentiation? Are their deacetylation functions redundant during the development of pistils and ovules? The author should provide the reason for studying HDA19 in these processes.

We have included in the revised manuscript a more detailed description of the rationale driving our choice, focusing on HDA19 (lines 148–155).

2. In the analysis of FACS coupled with RNA-seq, there are several upregulated genes were identified which supposed to be related to TT development. Why did the author only select STM for subsequent research (line 279) but not others, such as CLV1?

We agree with reviewer 2 that this could be better explained, and we have added a more detailed description to explain the rationale of the choice that prompted us to prioritize STM over the other upregulated meristematic regulators (lines 269–275). We also discuss this point in more detail in the discussion (see lines 424-439).

In addition, the hda19-3 STM RNAi line 1 and hda19-3 STM RNAi line 10 selected by the authors were not the lines with lowest STM transcription levels, but their complementary efficiencies of TT were the best among all lines. Is it possibly indicated that there is no direct relationship between STM transcription levels and TT development?

We agree with Review 2 that a more detailed explanation is required, and we have added it to the revised manuscript. See lines 315–316 and 332-334.

3. HDA19 does not directly bind to DNA, therefore it must interact with transcription factors or other cofactors to regulate its target. Before flower developmental stage 8, there are many transcription factors that can be candidates. Why did the authors directly use STK (line 370) as the only candidate and conduct genetic experiments?

We have added a more detail explanation of the rationale of choosing STK (see lines 367-374).

4. The most important point of this manuscript is the epigenetic regulation by HDA19 during ovule initiation and TT development. Since ChIP assay only reveals the binding of protein to DNA but not the acetylation, the conclusion of acetylation level is a little bit overstate. It supposed to be direct assay to detect the acetylation in hda19 mutant and WT.

We believe this is a misunderstanding. As stated in lines 286-291 we performed a ChIP-qPCR experiment using an antibody against acetylated histones (acH3K9): "To analyse whether the changes in *STM* expression pattern could be caused by variations in its histone acetylation levels resulting from the absence of HDA19, we collected flowers from stages 1-12 (excluding the IM) from *wild-type* and *hda19-3* plants and performed ChIP-qPCR. We used an antibody against HISTONE 3 (H3) acetylated in Lysine 9 and 14 (K9/14) (AcH3K9/14) (Figure 4I-J) to capture DNA fragments associated with acetylated histones".
Nevertheless, we have explained these experiments also in the material and methods (see lines 643-647).

5. Previous studies have shown that HDA19 interacts with ETT, ARF4, and FIL to regulate STM in IM (Chung et al., 2019), indicating that the function of HDA19 has been reported and HDA19 affects different processes of growth and development. HDA19 mutant has many abnormal phenotypes in the process of pistil development and double fertilization, such as influencing the initial number of ovules without affecting the pistil length. Why did the authors only study TT in this work (the authors mentioned ovule number determination in the title but focus on TT development in the manuscript).
We believe this is a misunderstanding, as s a significant part of the results is dedicated to the ovule number phenotype in the hda19 mutant (Figure 1B-D), in the complementing lines (Figure 5B-C), as well as that of the stk and stk shp1 shp2 mutants (Figure 6I-J).

Besides, the author's description of TT abnormalities was not clear enough. What is the conclusion, the ectopic proliferation of cells caused by STM overexpression (Figure 1 E-H, Figure 4 F-G), or the abnormal phenotype of TT caused by non-fusion septum (Figure S5 A-C), and are both related to the function of STM? The authors tried to include many points in one paper, which leads to unclear conclusion in each point.
We believe the reviewer mixed up ectopic expression with ectopic proliferation. What we have explained in our manuscript is that the ectopic expression of STM led to downregulation of genes involved in the differentiation process, as was shown for another meristem. In the text, 'ectopic proliferation' is not used, and it is unclear what the reviewer means by it.

6. This study focuses on the phenotype of TT by using pSTK: STK-GFP mediated FACS for RNA-seq. It is known that STK is expressed in the placenta before ovule initiation and in the ovules lately. Is STK expressed in TT? Figure S2 did not capture the STK fluorescence at TT, please double check it.
We have included a higher magnification of supplemental Figure S2. The objective of S2 is the comparison between STK::STK-GFP expression in wt with respect to the hda19 mutant. The STK::STK-GFP expression in wild-type TT and ovules was previously described in more than one manuscript, which we cited. Herrera-Ubaldo et al., 2019, Mizzotti et al., 2014, and Di Marzo et al., 2020.

7. Because the FACs coupled RNA-seq is a very precise experiment, the authors should describe the methods very clearly. In this article, the developmental stage of flowers collected for materials are very important. The authors described that they collected flowers at stages 1-12. However, according to experiences, the pistils of the 1-6 stages are very difficult to separate (barely to see). How did the author do that, directly take the entire inflorescence apex, or collected buds then remove other parts to leave the pistils?

It is not necessary to collect pistils using organ-specific cell sorting. We have added a more detailed description of the sampling and protoplast sorting process in the material and methods section (see lines 587–589).

There is no detailed description in the materials and methods. Please provide details in the section of sampling process. The figures are not finalized. The background of plants and the processing time are not labeled in almost all images. For example, in Figure 1, there are different developmental stage of ovules in the same placenta at flower developmental stage 9, which are not reflected in the model of ovule development. Are Figure 1B, C, E, F, G, H mutant or WT? As well as Figures 4 A-H, 6 A-H, S1, S2, etc. The readers only can find the information from brief legend.

Information regarding the different panels was available in the legend of the figures. We agree with reviewer 2 that this makes it necessary to read the legend to understand the figure, which might be inconvenient. We can add additional information to the panels (i.e., genotype, stage of development) to improve their readability.

In addition, the quality of many images in this article needs to be improved. The current images seemed not to be good enough and insufficient to gain conclusions. Please replace them with high-quality images. For example, the staining assay of pollen tube in Figure S1. Figure G-H showed an incomplete pistil, with the bottom cut off. The fluorescence intensity and white balance of each piece of image are different. The ovules on all placentae crowded and couldn't be seen clearly, suggesting the results and figures are preliminary.

The top panels of Supplemental Figure 1 (A, C, G, and H) show pistils where valves have been removed for clearer visualization of the growth of the pollen tubes. They are not cut, but as the valves have been removed, the bottom of the TT is straight, which might give the wrong impression that the pistil is cut. One can realize that this is not the case by checking the size of each pistil (they are all similar and the scale is the same) or by counting the ovules (the number of ovules present in each placenta is the typical number of the depicted genotype).Supplemental Figure 1 gives support to Figure 1I, where a graph showing the extent of pollen tube growth in the pistils of the different phenotypes was measured. The relevant information in this figure is the pollen tube growth, not the number of ovules.

02-Oct-2023

Prof. Lucia Colombo
Universita degli Studi di Milano
Biosciences
Milano 20133
Italy

RE: HDA19 CONTROLS OVULE NUMBER DETERMINATION AND TRANSMITTING TRACT DIFFERENTIATION BY REGULATING THE CMM ACTIVITY

Dear Lucia:

We have received reviews of your manuscript, which you may view at the end of this letter. On the basis of the advice received and my own evaluation, I believe that, pending revision, this article may ultimately be acceptable for publication in Plant Physiology.

While Reviewer #1 is satisfied with the revised manuscript, which adequately addressed all comments and suggestions, Reviewer #2 still raised a few concerns. However, in my opinion these appear mainly as misunderstandings and can be considered by partly better explaining the issues raised in the text. E.g. issue #1 regarding ChIPseq and acetylation pattern could be better explained in the results section instead of the methods section, issue #2 - reorganization of data - should be considered as suggested (or discussed) and issue #3 should considered, which requires a sentence explain the sampling of whole inflorescences. Please respond to all comments and improve the few minor points as well.

When you are ready to submit your revision, click the below link and find the red arrow under your Author Tasks heading for this revision submission.

If you want to visit this version of the manuscript, you may click the link below and then click on the folder that reads "Post Decision Manuscripts".

Link Not Available

When uploading the revised version of this article, please be sure to include as complete an answer as possible to the referees' criticisms during the first round of reviews in the "response to reviewers" section.

Plant Physiology considers a revision for up to 60 days after a manuscript has been provisionally accepted; limited extension of this timeframe is at the discretion of the Monitoring Editor. A reminder notice will be sent to you 2 weeks before the deadline, at which time you may choose to contact the editorial office to request an extension; if so, please provide a justification for your request. After the expiration of this deadline, the article would need to be submitted as a resubmission and would be handled as such.

Thank you very much for giving us an opportunity to review this work. I look forward to receiving the next version.

Sincerely,

Plant Physiology Board of Editors

Want to add this revision deadline to your calendar? Click below!

--- Reviewer comments:
Reviewer #1 (Comments for the Author):

I appreciate the authors' response to the previous comments and the efforts made to address them. They have provided a reasonable explanation for their focus on STM as a key regulator affected by HDA19 and why they didn't perform

complementation assays with other known regulators or downstream targets of HDA19 or STK. While additional functional evidence could have strengthened the study, the unique cell-specific transcriptome datasets still contribute significantly to our understanding of ovule development. The addition of a more detailed discussion of the proposed interaction between HDA19 and STK, including potential post-translational modifications or indirect regulation, is a welcome improvement. This enhances the mechanistic understanding of the regulatory processes discussed in the manuscript. Overall, I find that the authors have adequately addressed my comments and suggestions, improving the clarity and depth of their manuscript. This work continues to provide valuable insights into ovule development and the role of HDA19 in regulating this process.

Reviewer #2 (Comments for the Author):

This resubmitted manuscript "HDA19 CONTROLS OVULE NUMBER DETERMINATION AND RANSMITTING TRACT DIFFERENTIATION BY REGULATING THE CMM 2 ACTIVITY" addressed some of my questions for last version by adding some descriptions and reorganizing some figures. This revised manuscript looked more logically, which was better than the original version, although some results were still confused. The further modifications are still needed before consideration of publishing in the Plant Physiology. My concerns and suggestions are showed below.

1. The original comment: The most important point in this manuscript is the epigenetic modification by HDA19 during ovule initiation and TT development. Since ChIP assay only reveals the binding of protein to DNA but not the acetylation, the conclusion of acetylation level is a little bit overstate. It supposed to be direct assay to detect the acetylation in hda19 mutant and WT. The author used anti-acetylated histone 3 (H3) (AcH3K9/14) antibodies to indirectly represent the deacetylation process, which is confusing. According to the Figure S4, there are no other histone deacetylases significantly dysregulated in the selected cells of the hda19-3 mutant. Please explain in detail why selected AcH3K9/14 antibodies.
The authors' answer: We believe this is a misunderstanding. As stated in lines 286-291 we performed a ChIP-qPCR experiment using an antibody against acetylated histones (acH3K9): "To analyse whether the changes in STM expression pattern could be caused by variations in its histone acetylation levels resulting from the absence of HDA19, we collected flowers from stages 1-12 (excluding the IM) from wild-type and hda19-3 plants and performed ChIP-qPCR. We used an antibody against HISTONE 3 (H3) acetylated in Lysine 9 and 14 (K9/14) (AcH3K9/14) (Figure 4I-J) to capture DNA fragments associated with acetylated histones". Nevertheless, we have explained these experiments also in the material and methods (see lines 643-647).
New comment: What I mean is, ChIP assay is generally used to reveal whether the protein can bind DNA in vivo. There are other methods for detecting acetylation level directly. The similar point also came from "Editor comment".

2. The original comment: Previous studies have shown that HDA19 interacts with ETT, ARF4, and FIL to regulate STM in IM (Chung et al., 2019), indicating that the function of HDA19 has been reported and HDA19 affects the entire growth and development. HDA19 mutant has many abnormal phenotypes in the process of pistil development and double fertilization, such as influencing the initial number of ovules without affecting the pistil length. Why did the authors only study TT in this work? The authors mentioned ovule number determination in the title but focus on TT development in the manuscript. Besides, the author's description of TT abnormalities was not clear enough. What is the conclusion, the ectopic proliferation of cells caused by STM overexpression (Figure 1 E-H, Figure 4 F-G), or the abnormal phenotype of TT caused by non-fusion septum (Figure S5 A-C), and are both related to the function of STM?
The authors' answer: We believe this is a misunderstanding, as s a significant part of the results is dedicated to the ovule number phenotype in the hda19 mutant (Figure 1B-D), in the complementing lines (Figure 5B-C), as well as that of the stk and stk shp1 shp2 mutants (Figure 6I-J). We believe the reviewer mixed up ectopic expression with ectopic proliferation. What we have explained in our manuscript is that the ectopic expression of STM led to downregulation of genes involved in the differentiation process, as was shown for another meristem. In the text, 'ectopic proliferation' is not used, and it is unclear what the reviewer means by it.
New comment: What I mean is, it is not necessary to combine two different results (HDA19 functions in ovule initiation and TT development) together (no tight relationship between these two results and they could be two separated stories). Comparing to HAD19-STK regulating STM for TT development, the regulatory mechanism of HDA19 regulating ovule initiation is not detailed. Besides, the logic of results organization is not perfect. I prefer this way: First, the phenotypes of reduced seed set (fertilized ovules), which is easiest to be observed. Second, based on the Supplemental Figure S1, some pollen tubes couldn't turn to ovules. Is it possible that something wrong in female gametophyte development and pollen tube attraction, which might contribute to reduced seed set? Experiments or citations are needed to exclude the possibilities. If not, then transfer to male part. And limited pollination is much better than saturation pollination for statistical analysis of "ratio of pollen tube length/pistil length". Third, the defects of the germination ratio of pollen grain and elongation of pollen tube should be excluded (had19 has no effect in those processes, please cite literatures). Finally, gain the conclusion that HDA19 leads to the defective TT development.

3. The original comment: Because the FACs coupled RNA-seq is a very precise experiment, the authors should describe the methods very clearly. In this article, the developmental stage of flower collected for materials are very important. The authors described that they collected flowers at stages 1-12. However, according to experiences, the pistils of the 1-6 stages are very difficult to separate (barely to see). How did the author do that, directly take the entire inflorescence apex, or collected buds then remove other parts to leave the pistils? There is no detailed description in the materials and methods. Please provide details in the section of sampling process.

The authors' answer: It is not necessary to collect pistils using organ-specific cell sorting. We have added a more detailed description of the sampling and protoplast sorting process in the material and methods section (see lines 587-589).
New comment: I had a clerical error last time, "However, according to experiences, the pistils of the 1-6 stages are very difficult to separate (barely to see)" should be "However, according to experiences, the buds of the 1-6 stages are very difficult to separate (barely to see)." The first bud visible by eyes should be in stage 7-8. The authors added some description in the method section of the revised version "We collected flowers from whole inflorescences, leaving the meristem out, by cutting the flowers (stages 1-12) just below their base with tweezers. While including flowers in stages 1-7 might result in lower enrichment, it does not alter the result as STM and STK are not significantly expressed at those stages". It is difficult to image how to "cut the flowers (stages 1-6) just below their base with tweezers". Did the authors cut inflorescence apex for collecting buds in stage 1-6 under stereoscope? It would be helpful to show the images of buds with bar in stage 1-6. Otherwise, it is not necessary to mention those stages since the authors indicated 1-7 stages might result in lower enrichment.

Minor points:
1. Figure 1A, during flower developmental stage 9, the ovule primordia have protruded asynchronously and there are different sizes/shapes ovule primordia in the same placenta (Yu et al., 2020). The scheme of stage 9 should be modified. And the introduction section misses this information.
2. Figure 6E-H, the images labeled hda19-3, but the figure legend labeled stk shp1 shp2. Please correct the error.
3. Other small mistakes. For example, Supplemental Figure S1, "A-B) pollen tubes in a wild-type pistil at 12 HAP" should be "A-B) Pollen tubes in a wild-type pistil at 12 HAP".

First revision

Outline of author's responses to editor's and reviewer's comments (authors responses written in bold):

Editor comment:

"My major concern is, however, that the main conclusion that epigenetic regulation by HDA19 controls ovule initiation and transmitting tract differentiation via affecting the acetylation level appears not fully justified as acetylation levels were not investigated".

Acetylation ChIP of STM is indeed present in the manuscript. The acetylation level of *STM* in *hda19* with respect to the wild type has been analyzed (see Figure 4I-J). Acetylation levels in stk mutant were also analyzed (Figure 7A). We have added a sentence to emphasize the nature (lines 289-291 and 382-395) and importance (lines 302-305, 471-474) of these experiments.

-- Reviewer comments:
Reviewer #1 (Comments for the Author):

The manuscript entitled "HDA19 controls ovule number determination and transmitting tract differentiation by regulating the CMM activity" reveals the function of the HDA19 gene to downregulate SHOOT MERISTEMLESS (STM) in the carpel margin meristem (CMM), allowing ovule formation and transmitting tract (TT) differentiation. Overall, the manuscript provides valuable insights into the molecular mechanisms underlying ovule development and the regulatory roles of HDA19. The study addresses an important gap in understanding the genetic and molecular regulation of ovule number and transmitting tract development. The findings shed light on the roles of HDA19 in controlling ovule initiation and tissue differentiation, and their interaction in regulating gene expression patterns. The manuscript is well-organized, with a clear introduction, methods, results, and discussion sections. The figures and supplementary data provided support the findings and aid in the interpretation of the results. The logical flow of the manuscript enhances the readability and comprehension of the study.
Suggestions for improvement:

1. Although the authors demonstrate the upregulation of STM and other genes in hda19-3 mutant and the rescue of ovule defects by reducing STM levels, however, it would be valuable to include additional functional evidence, such as complementation assays with other known regulators or downstream targets of HDA19 or STK, to further support their conclusions.

Our manuscript reports unique cell-specific transcriptome datasets to identify new possible master regulators that must be silenced by HDA19 for correct transmitting tract development and ovule number regulation. These datasets are of considerable value to those studying developmental processes related to plant reproduction. To further support the importance of our datasets, we focused on STM, one of the few master regulators that was up-regulated in

the *hda19* mutant and whose up-regulation could explain the phenotype that we observed in the *hda19* mutant.

We agree with the reviewer that some other genes involved in transmitting tract development and ovule number regulation that we and other groups have already studied could be very interesting to include (Cucinotta et al., 2016; Cucinotta et al., 2018; Gomez et al., 2018; Herrera-Ubaldo et al., 2019; DiMarzo et al., 2020). However, the genes known to be important for ovule number determination and TT differentiation are downregulated in HDA19, suggesting that they probably are not HDA19 direct targets.

2. Are there other factors or regulators that might contribute to the phenotypes observed in hda19-3 mutant?

Yes, we indeed think this is the case. We have added a paragraph discussing this topic. For more details, see lines 424-439 and 445-450.

3. Consider rephrasing sentences 66-69 to improve clarity. The current phrasing may cause confusion regarding the relationship between ovule fertilization, seed formation, and the emergence of the reproductive tract (ReT) and ovules from the carpel margin meristem (CMM).

We have changed the sentence as suggested (see lines 65-68)

4. In the discussion the authors could provide a more detailed and mechanistic discussion of the proposed interaction between HDA19 and STK, such as potential post-translational modifications or indirect regulation.

We have included, as suggested by reviewer 1, a more detailed description of possible modes of interaction between HDA19 and STK. See lines 497-517.

5. The detail about the sample and stages of the flower used for the ChIP-qPCR should be included.

We have included a more detailed description of the stages of the flower used for the experiments, as suggested. See lines 643–647.

Reviewer #2 (Comments for the Author)

Comments:
The manuscript entitled "HDA19 CONTROLS OVULE NUMBER DETERMINATION AND RANSMITTING TRACT DIFFERENTIATION BY REGULATING THE CMM 2 ACTIVITY" studied HDA19 role in ovule number determination and ReT differentiation. hda19-3 mutant displays reduced ovule number and fails to properly differentiate the TT. The author used FACS coupled with RNA-seq to reveal that genes promoting organ development are downregulated in the CMM of hda19-3 mutant while meristematic markers (including STM) are upregulated, which means HDA19 downregulates STM in the CMM, allowing ovule formation and TT differentiation. STM is ectopically expressed in hda19-3 at intermediate stages of pistil development, and its downregulation by RNA interference alleviated hda19-3 phenotypic defects. ChIP indicated that STM is a target of HDA19 during pistil development and that STK is required for the histone acetylation-mediated regulation of STM. Basically, I considered that this work is important and interesting. The epigenetic regulation of ovule initiation is a good point. However, it seemed that the authors are too hurry to submit this manuscript. Many results and figures

are too preliminary, and the organization of this manuscript is not logically enough. The conclusions didn't include some important phenotypes and mechanisms, or, solve some essential questions. I suggested that this study could not been considered to publish in Plant Physiology until the authors should reorganize the manuscript and carefully address all the questions raised by reviewers and editor. The main issues are followed:

1. HDA19, HDA6, and other HISTONE DEACETYLASEs are reported to involved in the growth and development of plants, such as flowering, leaf formation, and embryonic development. However, the author directly selected HDA19 as the aim gene from the very beginning of this study, and only the HDA19 mutant was used for analysis. How about other HDAs? Do they also have similar phenotypes during ovule number determination and TT differentiation? Are their deacetylation functions redundant during the development of pistils and ovules? The author should provide the reason for studying HDA19 in these processes.

We have included in the revised manuscript a more detailed description of the rationale driving our choice, focusing on HDA19 (lines 148–155).

2. In the analysis of FACS coupled with RNA-seq, there are several upregulated genes were identified which supposed to be related to TT development. Why did the author only select STM for subsequent research (line 279) but not others, such as CLV1?

We agree with reviewer 2 that this could be better explained, and we have added a more detailed description to explain the rationale of the choice that prompted us to prioritize STM over the other upregulated meristematic regulators (lines 269–275). We also discuss this point in more detail in the discussion (see lines 424-439).

In addition, the hda19-3 STM RNAi line 1 and hda19-3 STM RNAi line 10 selected by the authors were not the lines with lowest STM transcription levels, but their complementary efficiencies of TT were the best among all lines. Is it possibly indicated that there is no direct relationship between STM transcription levels and TT development?

We agree with Review 2 that a more detailed explanation is required, and we have added it to the revised manuscript. See lines 315–316 and 332-334.

3. HDA19 does not directly bind to DNA, therefore it must interact with transcription factors or other cofactors to regulate its target. Before flower developmental stage 8, there are many transcription factors that can be candidates. Why did the authors directly use STK (line 370) as the only candidate and conduct genetic experiments?

We have added a more detail explanation of the rationale of choosing STK (see lines 367-374).

4. The most important point of this manuscript is the epigenetic regulation by HDA19 during ovule initiation and TT development. Since ChIP assay only reveals the binding of protein to DNA but not the acetylation, the conclusion of acetylation level is a little bit overstate. It supposed to be direct assay to detect the acetylation in hda19 mutant and WT.

We believe this is a misunderstanding. As stated in lines 286-291 we performed a ChIP-qPCR experiment using an antibody against acetylated histones (acH3K9): "To analyse whether the changes in *STM* expression pattern could be caused by variations in its histone acetylation levels

resulting from the absence of HDA19, we collected flowers from stages 1-12 (excluding the IM) from *wild-type* and *hda19-3* plants and performed ChIP-qPCR. We used an antibody against HISTONE 3 (H3) acetylated in Lysine 9 and 14 (K9/14) (AcH3K9/14) (Figure 4I-J) to capture DNA fragments associated with acetylated histones".
Nevertheless, we have explained these experiments also in the material and methods (see lines 643-647).

5. Previous studies have shown that HDA19 interacts with ETT, ARF4, and FIL to regulate STM in IM (Chung et al., 2019), indicating that the function of HDA19 has been reported and HDA19 affects different processes of growth and development. HDA19 mutant has many abnormal phenotypes in the process of pistil development and double fertilization, such as influencing the initial number of ovules without affecting the pistil length. Why did the authors only study TT in this work (the authors mentioned ovule number determination in the title but focus on TT development in the manuscript).

We believe this is a misunderstanding, as s a significant part of the results is dedicated to the ovule number phenotype in the hda19 mutant (Figure 1B-D), in the complementing lines (Figure 5B-C), as well as that of the stk and stk shp1 shp2 mutants (Figure 6I-J).

Besides, the author's description of TT abnormalities was not clear enough. What is the conclusion, the ectopic proliferation of cells caused by STM overexpression (Figure 1 E-H, Figure 4 F-G), or the abnormal phenotype of TT caused by non-fusion septum (Figure S5 A-C), and are both related to the function of STM? The authors tried to include many points in one paper, which leads to unclear conclusion in each point.

We believe the reviewer mixed up ectopic expression with ectopic proliferation. What we have explained in our manuscript is that the ectopic expression of STM led to downregulation of genes involved in the differentiation process, as was shown for another meristem. In the text, 'ectopic proliferation' is not used, and it is unclear what the reviewer means by it.

6. This study focuses on the phenotype of TT by using pSTK: STK-GFP mediated FACS for RNA-seq. It is known that STK is expressed in the placenta before ovule initiation and in the ovules lately. Is STK expressed in TT? Figure S2 did not capture the STK fluorescence at TT, please double check it.

We have included a higher magnification of supplemental Figure S2. The objective of S2 is the comparison between STK::STK-GFP expression in wt with respect to the hda19 mutant. The STK::STK-GFP expression in wild-type TT and ovules was previously described in more than one manuscript, which we cited. Herrera-Ubaldo et al., 2019, Mizzotti et al., 2014, and Di Marzo et al., 2020.

7. Because the FACs coupled RNA-seq is a very precise experiment, the authors should describe the methods very clearly. In this article, the developmental stage of flowers collected for materials are very important. The authors described that they collected flowers at stages 1-12. However, according to experiences, the pistils of the 1-6 stages are very difficult to separate (barely to see). How did the author do that, directly take the entire inflorescence apex, or collected buds then remove other parts to leave the pistils?

It is not necessary to collect pistils using organ-specific cell sorting. We have added a more detailed description of the sampling and protoplast sorting process in the material and methods section (see lines 587–589).

There is no detailed description in the materials and methods. Please provide details in the section of sampling process. The figures are not finalized. The background of plants and the processing time are not labeled in almost all images. For example, in Figure 1, there are different developmental stage of ovules in the same placenta at flower developmental stage 9, which are not reflected in the model of ovule development. Are Figure 1B, C, E, F, G, H mutant or WT? As well as Figures 4 A-H, 6 A-H, S1, S2, etc.

The readers only can find the information from brief legend.

Information regarding the different panels was available in the legend of the figures. We agree with reviewer 2 that this makes it necessary to read the legend to understand the figure, which might be inconvenient. We can add additional information to the panels (i.e., genotype, stage of development) to improve their readability.

In addition, the quality of many images in this article needs to be improved. The current images seemed not to be good enough and insufficient to gain conclusions. Please replace them with high-quality images. For example, the staining assay of pollen tube in Figure S1. Figure G-H showed an incomplete pistil, with the bottom cut off. The fluorescence intensity and white balance of each piece of image are different. The ovules on all placentae crowded and couldn't be seen clearly, suggesting the results and figures are preliminary.

The top panels of Supplemental Figure 1 (A, C, G, and H) show pistils where valves have been removed for clearer visualization of the growth of the pollen tubes. They are not cut, but as the valves have been removed, the bottom of the TT is straight, which might give the wrong impression that the pistil is cut. One can realize that this is not the case by checking the size of each pistil (they are all similar and the scale is the same) or by counting the ovules (the number of ovules present in each placenta is the typical number of the depicted genotype).Supplemental Figure 1 gives support to Figure 1I, where a graph showing the extent of pollen tube growth in the pistils of the different phenotypes was measured. The relevant information in this figure is the pollen tube growth, not the number of ovules.

Second revision

Editor letter

We have received reviews of your manuscript, which you may view at the end of this letter. On the basis of the advice received and my own evaluation, I believe that, pending revision, this article may ultimately be acceptable for publication in Plant Physiology.

While Reviewer #1 is satisfied with the revised manuscript, which adequately addressed all comments and suggestions, Reviewer #2 still raised a few concerns. However, in my opinion these appear mainly as misunderstandings and can be considered by partly better explaining the issues raised in the text. E.g. issue #1 regarding ChIPseq and acetylation pattern could be better explained in the results section instead of the methods section, issue #2 - reorganization of data - should be considered as suggested (or discussed) and issue #3 should considered, which requires a sentence explain the sampling of whole inflorescences. Please respond to all comments and improve the few minor points as well.

issue #1 regarding ChIPseq and acetylation pattern could be better explained in the results section instead of the methods section

regarding RNA-seq and STM acetylation analysis we have explained it more clearly in both the results and methods sections. Furthermore, we have added a reference about the use of ChIP to investigate acetylation state at a specific locus or/and at genomic level, together with a more accurate description:

<Line 295> "To analyse whether the changes in STM's expression pattern could be caused by variations in histone acetylation levels resulting from the absence of HDA19, we collected the inflorescence apex (including flowers up to stage 12 but excluding the IM) from wild-type and hda19-3 plants and performed ChIP-qPCR. ChIP-qPCR it is a widely used technique to reveal differences in the levels of specific histone modifications at a specific locus. (Petrella et al., 2020; Saleh et al., 2008). We used an antibody against HISTONE 3 (H3) acetylated at lysine 9 and 14 (K9/14) (H3K9/14ac) (Figure 4I-J) to capture DNA fragments associated with acetylated histone H3".

ChIP is a method to understand if at a specific locus a protein of interest (or a modified isoform of it) binds the DNA or where in the genome a specific protein binds the DNA (ChIP-Seq). It is the most used method to study the acetylation state of a histone at a specific locus. By using specific antibodies against an acetylated isoform of a histone we can access if this isoform is present at a specific locus and by comparing for instance mutant against wild-type we can compare the difference in occupancy (enrichment) of the protein isoform at a specific locus as we describe in our manuscript.

issue #2 reorganization of data - should be considered as suggested (or discussed)

We think that the results as presented in the manuscript are in line with the aim of our investigation since the manuscript focuses on the genetic interaction between STM, required to maintain the CMM at meristematic state, and genes like STK, NTT, SHP1 and SHP2 that are required to promote differentiation of the CMM into transmitting tract (TT) and ovule primordia. Therefore, it is important to investigate the effect of STM overexpression on both TT and ovule primordia specification.

Reviewer 2 would like us to include other possible phenotypic defects, like pollen germination, pollen tube attraction, seed development etc, however, these phenotypes as all the other pleiotropic effects observed in hda19 mutants, are not the focus of the research that we describe in our manuscript.

The developmental process mentioned by reviewer2 are of great interests and we may investigate them in the future however these will require the identification of other factors regulated by HAD19 responsible for male gametophytic development or/and gametophytic interaction and/or seed development and the study of the networks downstream those factors.

Issue #3 should consider, which requires a sentence explain the sampling of whole inflorescences

Indeed, we believe there is still a misunderstanding. We have explained that collecting the complete inflorescences will ensure that we have all stages represented, including those between stage 1 and 6. However, since STK, which is used as a selection marker for the cell sorting, is hardly expressed before stage

7, the sorting will mostly select cells starting from stage 7 (TT differentiation and ovule primordia initiation start after stage 7). In the material and methods section related to the protoplast recovery and FACS, we have rephrased this sentence as follows: *<Line 606> "we collected whole inflorescences, except for open flowers, directly in the solution used for protoplasting".* Regarding the sampling procedure for the CHIP-qPCR experiments, in the material and methods we rephrased as follows*: <Line 662> "We collected the whole inflorescences leaving the IM out, by cutting the flowers (up to stage 12) just below their base with tweezers".*

minor points:

1. Figure 1A, during flower developmental stage 9, the ovule primordia have protruded asynchronously and there are different sizes/shapes ovule primordia in the same placenta (Yu et al., 2020). The scheme of stage 9 should be modified. And the introduction section misses this information.

To address the first minor point of the reviewer 2 we have added the following sentence in the introduction section: <line 88> *"Particularly, OP emerge from the placenta asynchronously. Indeed, ovules start to emerge from the placenta at stage 9a, while the final ovule number is reached only at stage 9c. This results in the presence of ovules of different sizes and developmental stages within the same pistil (Yu et al., 2020)".*

Concerning the scheme of in FIGURE 1A, the cross-section of the pistil at stage 9 already shows ovule primordia with different size.

2. Figure 6E-H, the images labeled hda19-3, but the figure legend labeled stk shp1 shp2. Please correct the error.

We are sorry for the error. We have modified the labeling as suggested.

3. Other small mistakes. For example, Supplemental Figure S1, "A-B) pollen tubes in a wild-type pistil at 12 HAP" should be "A-B) Pollen tubes in a wild-type pistil at 12 HAP".

We have corrected the mistake.

29-Oct-2023
Prof. Lucia Colombo
Universita degli Studi di Milano
Biosciences
Milano 20133
Italy

MSID: PP2023-RA-01337DR1
MS TITLE: HDA19 CONTROLS OVULE NUMBER DETERMINATION AND TRANSMITTING TRACT DIFFERENTIATION BY REGULATING CMM ACTIVITY

Dear Lucia:

I am pleased to inform you that your revised manuscript "HDA19 CONTROLS OVULE NUMBER DETERMINATION AND TRANSMITTING TRACT DIFFERENTIATION BY REGULATING CMM ACTIVITY" has been accepted for publication in Plant Physiology. The manuscript has been further improved and misunderstandings clarified.

Your manuscript will now be checked for consistency with journal formatting and image standards by one of our Science Editors, who will contact you shortly. They may have a number of queries and suggestions for you. Please keep in mind that they work to increase the online visibility of your article and its attraction for readers from the widest of backgrounds. You will have the opportunity to review any revisions, and I hope you will work constructively with them to get the most out of your research. You will also have the opportunity to review all changes when you receive the author proofs for your article.

Once validated for publication, your article will be sent to a Science Editor for a final review of your manuscript and source files. After your final files are uploaded, your article will appear in the next available issue and the online Preview version of your article will be posted after your signed Copyright and Checklist forms and final source files are received.

Please be sure to include the following in your manuscript file:
Harvard style citations
List of all author contributions and funding information at the beginning of manuscript after the titles and author list.
List all supplemental materials associated with the article, with titles, at the end of the ms, before the references and acknowledgments section.
Complete Figure Legends for all figures appearing in the article listed before the Literature Cited.

Thank you and congratulations on your Plant Physiology paper!

Sincerely,
Plant Phys Board of Editors

=====
FIRST AUTHOR PROFILES
=====

We recognize the hard work that goes into being First Author of a Plant Physiology paper by publishing a "First Author Feature" for each First Author alongside your paper.

If the first author(s) would like to submit information for a feature on Facebook and Twitter, fill out the form by clicking on the link below:

Plant Physiology First Author Feature Form: https://forms.office.com/r/ZBCGZfhm8F
If you cannot access the form through the link above, please contact Rachel at rbelsky@aspb.org.

To upload an image of yourself to accompany your profile, please put your photo in the correct journal folder at the link below. You must label your image: Author First Name Last Name Article Title.

First Author Profile Image Dropbox: https://www.dropbox.com/scl/fo/xz029p7qjr2jml2rp2kmq/h?

dl=0&rlkey=6fkxcaqmxx35ap152b2osy1fz

=====
IMPORTANT REMINDER: PEER REVIEW REPORTS
=====

If you opted to publish a peer review report along with your article during the original submission process, it will be prepared by the editorial staff and publicly posted with your manuscript, inside the zip file that contains any other supplemental material. As a reminder, the peer review report is a public record of all comments from editors and reviewers, as well as your prior responses, as you received them in the decision letters for each draft of your manuscript. If you agreed to publish this report and have changed your mind, or are not sure if you selected this option, please contact the editorial office as soon as possible before signing the license agreement from our publisher.

-- Reviewer comments:
